# Interpretable machine learning for hikikomori screening: The adaptive HRI-15

**DOI:** 10.1371/journal.pone.0355595

**Published:** 2026-08-20

**Authors:** Daiana Colledani, Pasquale Anselmi, Lucia Monacis, Bruno Genetti, Daniele Fassinato, Luis J. Gomez Perez, Adele Minutillo, Luisa Mastrobattista, Claudia Mortali

**Affiliations:** 1 Department of Psychology, Faculty of Medicine and Psychology, Sapienza University of Rome, Rome, Italy; 2 Department of Philosophy, Sociology, Education and Applied Psychology (FISPPA), University of Padova, Padua, Italy; 3 Department of Humanities, University of Foggia, Foggia, Italy; 4 Explora Research and Statistical Analysis, Padua, Italy; 5 Novella Fronda Foundation, Padua, Italy; 6 National Centre on Addiction and Doping, Italian National Institute of Health, Rome, Italy; Universita degli Studi di Pisa, ITALY

## Abstract

Hikikomori, or prolonged social withdrawal, is an issue of global relevance. The HRI-15 is a brief tool for its assessment. This study enhances its utility by adding clinical thresholds to identify risk and person-centered clinical profiles, and by developing a machine-learning(ML)–based computerized adaptive test (CAT) to enable rapid screening. Data from a national survey of Italian adolescents (*N* = 8,755) were used to conduct ROC analysis and latent profile analysis (LPA). The findings indicated that a score of ≥ 42 achieved optimal classification performance, and four profiles with distinct meanings and systematic differences in anxiety, depression, impulsivity, and risk behaviors were identified. A multivariate conditional inference tree was estimated to develop an ML-based adaptive version of the instrument. The CAT reduced administered items by 53% (7.03/15), accurately reproducing full-length scores (*r* = .77–.997) and the corresponding classification; alignment was assessed with latent transition analysis (entropy = .96). The procedure was integrated into an application supporting administration, scoring, and reporting (score, risk, profiles, graphs). Combining a cross-validated cutoff and clinically useful profiles with CAT and its automated administration and reporting application strengthens triage and personalization, enabling large-scale, multidomain screening and earlier intervention for hikikomori risk.

## Introduction

In recent years, the phenomenon of prolonged social withdrawal, known as hikikomori, has become a major challenge for mental health, education, and healthcare systems [1]. Hikikomori refers to a persistent pattern in which individuals substantially limit social participation and relationships outside the immediate family, remaining confined to their room or home for a long time [2–5]. In its broader sense, the term denotes a significant and problematic form of prolonged social withdrawal. Although hikikomori has been discussed in contemporary nosological debates and is mentioned in the DSM-5/DSM-5-TR [6] in relation to cultural concepts of distress, it is not listed as a discrete DSM-5/DSM-5-TR disorder. Rather, it is better understood as a clinically relevant and culturally inflected syndrome/condition whose nosological status remains under debate. Proposed research criteria typically emphasize marked home-based social withdrawal lasting at least six months, together with functional impairment or distress.

Although originally described in Japan in connection with school absenteeism (futoko) under intense educational demands [3,4,7–9], the phenomenon now exhibits a global spread across different age groups, with reported cases from Asian and Western settings, including the USA and EU [7,10–19]. This trajectory accelerated during the COVID-19 pandemic, which imposed extended periods of isolation and shifted social interaction and work into digital environments [20–22].

Despite the absence of unified global epidemiological estimates, available research suggests that hikikomori affects about 1.2% of the world population [17]. In Japan, studies have reported prevalence between 0.9% and 2.4%, with some studies indicating 22% in specific samples of young people [2,5,15,23,24] Estimates of similar magnitude emerge in other Asian contexts: Hong Kong 5.0–9.10% [25,26], mainland China 1.90–6.57% [27–29], and Korea around 1.4% [30]. In Western countries, estimates range from 2.03% to 16.13% in the EU [31,32] and are about 2.65% in the United States [7]. In general, prevalence figures vary widely across studies due to differences in cultural context, target population, and measurement tools; for example, a national survey in Oman found rates close to 44%, markedly surpassing estimates from other countries [33]. In Western countries, although systematic surveillance remains limited, converging evidence points to a substantial problem, especially among young people. Consistent with this, a national study on Italian adolescents showed that social withdrawal tendencies are already present at age 13, underscoring the need for targeted early detection [34]. The literature further indicates that, even though onset typically occurs in adolescence [20,24,35,36], once established, withdrawal often persists over the years, with detrimental consequences for personal development, academic/occupational functioning, and psychological well-being [16,19,24,37–41]. Taken together, these findings underscore the need for early identification and large-scale screening to limit chronicity and enable timely interventions.

To address this need, psychometric research has developed specialized screening instruments. Among the most widely used self-report measures are the NEET-Hikikomori Risk [42], the Hikikomori Questionnaire [43], and the Hikikomori Risk Inventory [44]. The HRI-24, developed jointly by Japanese and Italian researchers, shows robust psychometric properties and cross-cultural invariance, supporting its use as a promising instrument for international research on hikikomori. Moreover, its five-factor structure (anthropophobia, agoraphobia, lethargy, paranoia, and depressive mood) allows for the evaluation of distinct facets of the hikikomori condition. This is noteworthy because it may enable the creation of specific risk profiles, which would be clinically valuable as they could have unique antecedents and outcomes and inform personalized preventive and therapeutic pathways. Another strength of the instrument comes from the availability of a brief version, which facilitates its use in broad-spectrum screening and epidemiological studies. The brief form (HRI-15) has demonstrated adequate psychometric properties, including age- and gender-invariance, as well as ROC-derived cut-off scores showing sufficient diagnostic accuracy [11].

Although the instrument is valid and relatively time-efficient, it can still be challenging to use when multiple psychopathological conditions must be screened at once. In clinical practice, research surveys, and epidemiological studies, comprehensive assessments routinely require broad-spectrum screening across several health domains [45,46]. These procedures are essential for targeting interventions and describing populations and phenomena under study. However, administering extensive questionnaires increases testing time and respondent burden, raising the risk of fatigue, incomplete responses and reduced data quality [46–48]. These demands can be especially challenging for vulnerable individuals and require significant resources from service providers [45,49].

Computerized Adaptive Testing (CAT) offers a useful strategy to mitigate these issues. By tailoring item administration to prior responses, CAT markedly reduces the number of items while preserving score precision [45,50,51]. In recent years, integration with machine learning (ML) has made CAT procedures particularly efficient, accurate, and straightforward to implement. ML-based CAT can reduce administration burden without compromising validity, maintain high classification accuracy, and deliver reliable clinical categorizations, including in longitudinal settings [52–54].

This study aims to introduce an ML-based computerized adaptive version (ML-based CAT) of the HRI-15 that shortens administration and reporting time while improving classification reliability and clinical utility. The procedure automatically returns dimensional scores, flags scores that exceed the ROC-based cut-off for hikikomori risk, and assigns respondents to previously defined risk profiles (e.g., via latent profile analysis). By providing faster, more accurate, and practice-ready outputs, the ML-based CAT has the potential to enhance the accuracy and efficiency of clinical, scientific, and epidemiological assessments and screening procedures.

### Aims and analytic overview

To meet the proposed objectives, the study advances through two principal stages: first, the definition of detailed diagnostic criteria and clinical risk profiles; second, the development of an ML-based CAT version of the instrument with an automated administration and reporting application.

As mentioned above, the definition of specific risk profiles based on HRI dimensions is expected to be particularly valuable, as it has the potential to identify specific risk groups, allowing for the analysis of their distinctive characteristics and associated factors. This, in turn, should lead to the development of more personalized and targeted interventions, fully leveraging the tool’s potential. In parallel, the development of an ML-based CAT version of the tool has the potential to reduce respondent boredom and fatigue thereby increasing enrollment rates and survey adherence, facilitating longitudinal monitoring, simultaneously reducing time, costs, and scoring errors.

### Diagnostic categorization

To be diagnostically useful, test scores must be translated into explicit decision criteria. In this regard, this study aims to make two primary contributions: the establishment of a cut-off score for identifying hikikomori risk using receiver operating characteristic (ROC) analysis; and the delineation of latent profiles derived from the HRI-15 dimensions through latent profile analysis (LPA).

Establishing a cut-off score for classifying at-risk individuals is critical to enhancing the practical value of any screening or diagnostic instrument. Operationally, ROC analysis provides a rigorous procedure for determining, among all possible test scores, the threshold that most effectively differentiates individuals with and without a specific condition [55]. ROC analyses require two inputs: observed scores on the target measure and an external criterion used as a pragmatic indicator of whether cases meet the target condition. For each possible score, sensitivity (true positive rate) and specificity (true negative rate) are estimated, and the optimal cut-off score is selected as the score that maximizes both [55]. In this study, consistent with the literature indicating a general factor underlying the HRI-15, the total scale score was used as the target measure to establish the optimal cut-off score for the scale [11]. The external criterion was a self-report item assessing how often participants locked themselves in their rooms during the past six months. Responses were recorded on a frequency scale and dichotomized as not at risk (“Never”, “Only once”, “1–2 times per month”) or at risk (“Every week (but not every day)”, “Every day”). This item was considered appropriate for calibrating a practical screening threshold because it directly reflects a core behavioral manifestation of prolonged social withdrawal. Previous work on the HRI-15, using a similar procedure, identified a cut-off score (≥ 37) with encouraging performance (i.e., AUC = 0.80; sensitivity = 0.78; specificity = 0.71; accuracy = 0.72; [11]); however, those analyses relied on a binary external criterion (“Have you ever experienced the tendency to lock yourself in your room for several months, never going out, not even to eat meals or to entertain social relations?”; yes/no). The present study advances this evidence by using a larger sample and a more detailed, frequency-based criterion, thereby enabling a more behaviorally specific cut-off calibration and a more detailed assessment of classification performance.

While establishing precise cut-off scores is crucial for clinical practice, these values are confined to reflecting only the total score. Although suitable for preliminary screening, this approach overlooks potentially meaningful profile differences, thereby limiting the development of individualized interventions and the advancement of understanding of the phenomenon. To address this limitation, the present study proposes a novel contribution through LPA. LPA is a person-centered, model-based approach that partitions a population into latent groups characterized by within-class homogeneity and between-class heterogeneity on a set of indicators [56,57]. It is a rigorous method that yields clinically meaningful information, which in turn can facilitate the development of more individualized prevention and treatment approaches [58,59]. Specifically, in this work, LPA was used to detect latent profiles based on the five HRI-15 dimensions. The emerging profiles were also examined against a set of psychosocial indicators (e.g., anxiety, depression, impulsivity, substance use, and related functional outcomes) to assess their clinical utility. Notably, this profile-based characterization has not yet been undertaken for the HRI; however, it has the potential to substantially expand the instrument’s utility by fully leveraging its value for screening and interventions.

### CAT development

In clinical and research contexts, assessment batteries often incorporate multiple instruments to gain a nuanced understanding of individuals and populations. While this approach provides valuable insights, it also increases the workload for respondents, professionals, and organizations. The use of brief scales can be an effective strategy for managing this issue. However, when many constructs need to be evaluated, the overall assessment length can still be quite extensive, even if each construct is measured with a brief scale. CAT is an innovative and effective method for improving measurement efficiency without compromising accuracy [50,51]. Computerized adaptive tests (CATs) personalize the testing experience by presenting respondents with a reduced number of items selected dynamically for each person based on item properties and the responses progressively provided by participants during the testing process. Consequently, unlike fixed-form tests, CATs do not administer the entire set of items to each respondent, but focus on a restricted set of items that are most informative for each respondent at a given time [60,61]. Most CATs originate in the framework of item response theory (IRT) and have demonstrated notable efficiency in in various fields, including education, mental health and personality assessment, as they facilitate the precise measurement of disorder severity using a markedly limited number of items [62–66].

More recent approaches to CAT development draw on ML algorithms based on recursive binary partitioning, such as Classification and Regression Trees (CART). Compared to IRT-based procedures, these methods are generally easier to implement because they do not require calibration of large item banks and adherence to strict distribution or dimensionality assumptions (e.g., unidimensionality). Furthermore, their straightforward integration into administration systems and provision of transparent, clinically relevant decision rules enhance their utility and appeal in psychological and psychodiagnostic settings [34,46,52–54,67,68]. Early work in this area focused on procedures that assigned respondents to discrete categories (e.g., diagnosed vs. not diagnosed; at risk vs. not at risk). This framework is known as computerized adaptive diagnosis (CAD; [46]). Recently, however, CART-based approaches have been extended to estimate continuous outcomes, such as test scores, disorder severity, and trait levels. This enables a more nuanced quantitative assessment that goes beyond simple categorization, thus expanding their range of applications [53,67,69].

In general, CART algorithms operate by developing a tree structure that can be conceptualized as a flowchart defined through a recursive, top-down, divide-and-conquer process that generates a sequence of if-else rules aimed at creating classifications (decision trees, DT) or continuous predictions (regression trees, RT; model trees, MT; [70,71]). The tree comprises a root (the variable from which prediction/classification begins), internal nodes (additional variables considered along the path), branches, and leaves. Branches are constituted by a sequence of nodes, connected through specific decision rules (e.g., a numerical threshold of ≤ 1 vs. > 1 for a 0–5 variable) and leaves correspond to the termination of a branch, representing the final prediction of the defined process (class or score).

As binary partitioning methods, CART algorithms split cases according to the rules coded in the tree structure. The development of the algorithm starts with the selection of a root variable that includes the entire dataset, as well as an associated splitting rule that divides the sample into subsets that are as homogeneous as possible with respect to the outcome (classification or score). The procedure then continues recursively within each subset until specific stopping criteria are met (e.g., maximum depth, minimum node size, or no further gain in homogeneity; [71]). In the context of categorical outcomes, the objective is to maximize node purity, a concept that is typically quantified via information gain and entropy reductions [71–74]. In the context of continuous outcomes, the implementation of splits is based on the objective of minimizing within-node variance. This is frequently operationalized as a reduction in standard deviation [71].

Model definition typically follows a two-step approach. First, the tree is developed using a dataset (training dataset) that contains predictors and outcomes for all cases. Then, its predictive performance is evaluated using an independent dataset (testing dataset) with the same structure, but comprising cases that were not used in the development stage [75,76]. This cross-validation approach provides an estimate of generalizability and practical utility of the learned tree for predicting unseen data.

An emerging body of research indicates that tree-based adaptive procedures are effective in psychometric and diagnostic applications [46,53,67–69,77]. Conceptually, once a tree structure has been developed, it can be used to guide the adaptive administration of tests. Respondents are presented only with items located along the branch to which they are progressively assigned based on their responses [46,52,69,77,78]. This architecture offers several practical advantages. First, the adaptive process employs a precompiled scheme based on the established branching structure, thus allowing for rapid deployment with minimal runtime computation [68]. This contrasts with many IRT-based implementations, which require more complex procedures [60,61]. Second, this approach is less dependent on stringent theoretical assumptions, facilitating its application across clinical, school, and epidemiological contexts [46,54].

Evidence indicates that ML-based CATs perform well for both categorical classification and continuous score estimation, yielding valid and time-efficient assessments that closely align with full-length measures in cross-sectional and longitudinal designs [53,54,69,77–80]. Moreover, agreement with external variables is typically indistinguishable from that obtained with full-scale administrations [53,67].

Building on these foundations, the present study aims to demonstrate that an ML-based CAT can improve psychometric screening and diagnostic assessment by producing clinically meaningful outputs (accurate score estimation, risk-profile categorization, and precise cutoff-based classification) while simultaneously reducing respondent burden and facilitating test administration. This efficiency has the potential to enable large-scale, multi-domain screening, making administration more feasible in real-world settings without compromising accuracy or interpretability. In this context, the HRI-15 is an appropriate test case because it assesses distinct dimensions relevant to hikikomori, allowing the identification of clinically useful risk profiles that can substantially complement the essential cutoff-based classification that remains fundamental for screening. The manuscript details the development of an ML-based CAT and introduces a dedicated administration, scoring, and reporting application that returns dimensional scores in real time, flags cutoff exceedances, assigns LPA-based profiles, and generates a concise, practitioner-oriented report suitable for clinical, educational, and epidemiological contexts.

## Method

### Participants

Data were drawn from a large, nationally coordinated, school-based survey of Italian adolescents aged 11–17 years. The sampling plan was designed to produce a representative student cohort across the country and employed probabilistic selection with stratification by age band (11–13; 14–17), school type, and geographical area. To achieve at least 4,000 valid questionnaires per age group—and assuming a response rate of 15–20%—676 schools across all Italian regions were invited. Final participation rates were 22.7% for middle schools (ranging 20.0–26.2% across areas) and 22.6% for high schools (range 18.5–26.8%). All regions contributed data, ensuring broad national coverage.

In total, 10,181 questionnaires were submitted. Following data-quality checks, 1,426 records (14.0%) were excluded due to ineligibility (age outside target range), incomplete responses, or duplicate submissions caused by technical issues during online entry. The final analytic dataset thus included 8,755 valid cases.

Written informed consent was obtained from students and their legal guardians prior to participation. Data collection and processing complied with national privacy regulations, guaranteeing respondent anonymity. The study received ethical approval from the National Ethics Committee of the Italian National Institute of Health (Protocol PRE BIO CE 0010655, March 22, 2022) and adhered to the principles of the 1964 Declaration of Helsinki and its subsequent amendments.

The final sample comprised 8,755 students (*M* age = 14.03 years, *SD* = 1.98). Of them, 3,623 were middle-school students (41.4%; *M* age = 11.99, *SD* = 0.81) and 5,132 were high-school students (58.6%; *M* age = 15.48, *SD* = 1.08). Within the high-school subsample, 7.7% attended fine-arts secondary schools, 31.8% vocational institutes, 29.4% technical institutes, and 31.1% other types of high schools. The gender distribution was approximately balanced (4,187 females, 47.8%; 4,291 males, 49.0%), with 277 students (3.2%) not disclosing gender. Most participants reported Italian nationality (*N* = 7,535; 86.1%).

### Measures

All measures were collected using closed-ended items through a computer-assisted web interviewing platform during school hours. Trained supervisors were present to oversee the process. The battery comprised: demographic indicators (age, gender, region), lifestyle and behavioral indicators (e.g., participation in social-media challenges, alcohol use, cannabinoids use), and standardized scales assessing hikikomori risk and related psychosocial constructs.

#### Lifestyle and behavioral indicators.

One item assessed the tendency to self-isolate over the past six months (“Over the last six months, have you isolated yourself in your room for extended periods of time without ever going out—not even to eat meals or engage in social interactions?”). The response options were: 1 = Never, 2 = Only once, 3 = One to two times per month, 4 = Every week (but not every day), and 5 = Every day. For the analyses, the responses were dichotomized as not-at-risk (answers from 1 to 3) and at-risk (answers from 4 to 5).

Daily use of social media and video games over the previous week was assessed using two separate items. Participants were asked to quantify their usage by answering the following questions: “In the past week, how many hours per day did you use social media?” and “In the past week, how many hours per day did you play video games?” Responses were recorded on a five-point scale: 1 = Less than 1 hour, 2 = 1–2 hours, 3 = 3–6 hours, 4 = 7–8 hours, 5 = 9 hours or more.

An additional set of dichotomous (yes/no) items was administered to assess various risk behaviors. The following variables were included in the study: participation in social media challenges during the past six months (“In the past six months, have you taken part in social media challenges?”); history of alcohol consumption (“Have you ever drunk alcohol?”); history of sending erotic/sexual messages (i.e., sexting, “Have you ever sent erotic/sexual messages, videos, or personal photos via smartphone, e-mail, or webcam?”); and history of cannabis use (“Have you ever used cannabis?”; for ethical reasons the last two questions were presented only to the high school students group).

As a protective behavior, the tendency to seek assistance from parents or caregivers was measured using a yes/no item (“When you have problems, do you talk with your parents/caregivers?”).

#### Hikikomori risk.

Hikikomori risk was assessed with the Hikikomori Risk Inventory–15 (HRI-15; [11]). The scale is the short form of the HRI-24 [44] and comprises 15 items rated on a five-point Likert scale (1 = Strongly disagree; 5 = Strongly agree), referring to the past six months. The instrument indexes five dimensions: anthropophobia, agoraphobia, paranoia, lethargy, and depressive mood. These dimensions were derived through factor-analytic refinement from items tapping hikikomori symptomatology and related features as theorized by [8]. Anthropophobia, reflects fear of people and social contact (e.g., “I feel severely anxious when I meet people outside”); agoraphobia denotes avoidance of settings where assistance might be difficult to obtain during panic or intense anxiety (e.g., “I avoid enclosed public spaces such as shops, cinemas, theaters, or banks”); lethargy captures low energy and behavioral disengagement (e.g., “I sleep many hours a day because I often feel weak”); paranoia reflects suspiciousness and mistrust (e.g., “I am afraid of being deceived by others”); and depressive mood indexes sadness, anhedonia, and discomfort (e.g., “I feel a sense of inner emptiness”). Despite the inherent multidimensionality of the structure of the scale, existing evidence suggests that a robust general factor underlies the unidimensional interpretation of the total score [11]. The short form demonstrated satisfactory psychometric properties, with an area under the curve (AUC) of .80, sensitivity of .78, specificity of .71, and accuracy of .72. It also exhibited invariance by gender and age group (middle school versus high school). In this sample internal consistency was α = .92 for the total scale score, while it was .84, .83, .77, .82, and .79 for anthropophobia, agoraphobia, paranoia, lethargy, and depressive mood, respectively.

#### Related psychosocial constructs.

Social anxiety and depressive symptoms were assessed using the Italian adaptations of the DSM-5 Severity Measures for Social Anxiety [81] and Depression [82]. The social anxiety scale includes 10 items rated from 0 (“never”) to 4 (“all the time”) referring to the past seven days (e.g., “In the past seven days, I spent a lot of time thinking about what to say or how to behave in social situations”). The depression scale comprises 9 items rated from 0 (“not at all”) to 3 (“nearly every day”) referring to the past week (e.g., “In the past seven days, how often did you feel down, depressed, irritable, or hopeless?”). Higher total scores indicate greater symptom severity; internal consistency was α = .93 for social anxiety and α = .89 for depression.

Impulsivity was included as a divergent construct because, in the HRI-24 validation, sensation seeking (e.g., BSSS) was employed to assess divergent validity; sensation seeking is closely related to impulsivity, and both reflect approach-oriented, externalizing tendencies that stand in contrast to social withdrawal, the core of hikikomori [44,83–86]. Trait impulsivity was measured with the Italian BIS-15 (Barratt Impulsiveness Scale–15; [87]), a brief form of the BIS-11 [88]. Although the BIS-15 has not been specifically validated in Italian adolescents, the full version has been adapted for this population [89]. Items are rated on a four-point scale (e.g., “I have self-control”). Following recent recommendations [90], BIS-15 scores were treated as a continuous score; higher scores indicate greater impulsivity (α = .77).

## Analysis strategy

### ROC analysis.

ROC analyses were conducted on the HRI-15 total score using an external criterion based on the item: “Over the past six months, have you stayed in your room for extended periods without going out—not even for meals or social interactions?”. Responses were dichotomized as positive (“Every week, but not every day” or “Every day”) versus negative (“Never” through “One to two times per month”). The dataset was randomly partitioned into a training set (approximately 67% of the total sample) and a test set (remaining 33%). The training set (*n* = 5,866; 48.9% male; *M* age = 14.00, *SD* = 1.99) was used to estimate the ROC curve, compute the area under the curve (AUC) with its 95% confidence intervals, and determine the optimal cut-off via Youden’s index (Sensitivity + Specificity – 1; higher values indicate better combined sensitivity and specificity). The selected threshold was then applied to the independent test data set (*n* = 2,889; 49.3% male; *M* age = 14.10, *SD* = 1.96), to compute sensitivity, specificity, and overall accuracy with their 95% confidence intervals. ROC analyses were performed in the statistical environment R [91] using the pROC package [92]. To examine the robustness of the threshold to alternative operationalizations of the external criterion, sensitivity analyses were conducted using different dichotomizations of the room-confinement item. Specifically, the primary coding defined positivity as responses 4–5 (“Every week (but not every day)” / “Every day”), whereas a stricter coding treated only the response “Every day” as positive. This comparison was used to assess whether the resulting cut-off remained stable under a narrower definition of social withdrawal. Finally, supplementary calibration analyses were conducted by estimating logistic regression models in the training dataset and applying them to the testing dataset to obtain predicted probabilities. Calibration was evaluated by comparing predicted risk probabilities with observed outcomes in the testing dataset. The Brier score was used as a summary index of the overall discrepancy between predicted probabilities and observed outcomes, with lower values indicating better probabilistic accuracy [93]. Calibration intercept and calibration slope were also examined: the ideal values are 0 for the intercept and 1 for the slope; negative or positive intercept values indicate systematic overestimation or underestimation of risk, respectively, whereas slopes below or above 1 indicate overly extreme or overly moderate predicted probabilities, respectively [94].

### Latent Profile Analysis (LPA).

LPA was employed to detect latent profiles based on the five HRI-15 scales. LPA is a person-centered approach that classifies individuals into groups defined by comparable response patterns on a set of indicators, providing a holistic view of individual differences that can be especially informative in applied research and clinical practice [95–99]. To date, no latent-profile solution for the HRI-15 has been reported, making this analysis a novel contribution. The analysis was therefore conducted on the full sample to maximize precision and obtain realistic parameter estimates. In particular, a sequence of one- through five-class models was estimated and compared, using standardized scores on the five HRI-15 scales as indicators. To identify the preferred solution, multiple statistical and substantive criteria were considered jointly, including Akaike’s Information Criterion (AIC), the Bayesian Information Criterion (BIC), the sample-size–adjusted BIC (SABIC), and entropy. Entropy is a measure of classification accuracy ranging from 0 to 1. Higher values indicate a clearer separation between classes. Conversely, for AIC, BIC, and SABIC greater accuracy is suggested by lower values [57]. Two comparative tests were also considered for model evaluation: the Vuong–Lo–Mendell–Rubin likelihood ratio test (VLMR; [57]) and the Lo–Mendell–Rubin likelihood ratio test (LMR; [100]). These tests are used to compare a model with C latent classes against a model with C–1 classes. Significant *p*-values indicate that the model with C classes better fits the data than a more parsimonious one (i.e., the model with one fewer class). In contrast, non-significant *p*-values suggest retaining the more parsimonious model [57,100]. Finally, class sizes and the interpretability of the resulting profiles were taken into account in selecting the solution to be retained. As mixture-model fit indices often fail to converge on a single optimal solution, no single statistic was considered to be conclusive. Instead, model selection was based on an overall balance of relative model fit, classification quality, parsimony, class size, and interpretability [57,93,97]. Analyses were conducted using Mplus 7.4 [56], with the robust maximum likelihood estimator (MLR; [101]) and mixture specification. Between-class differences on a set of psychosocial indicators were examined using ANOVA and χ^2^ statistics, with eta-squared (η^2^) and Cramer’s *V* as effect size measures.

### Machine-Learning Computerized Adaptive Test (CAT).

**Fig 1:**
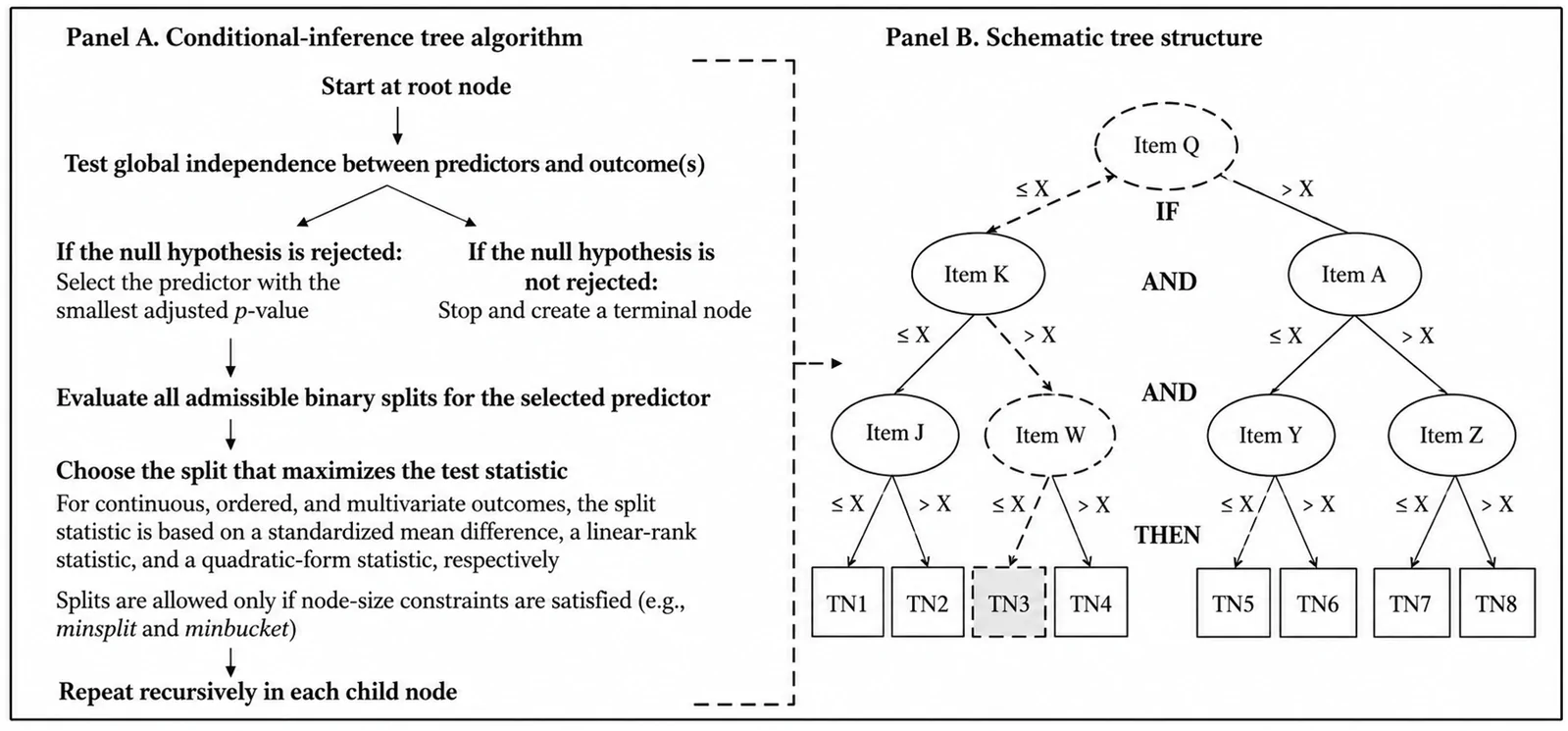
Overview of the conditional-inference tree procedure and schematic illustration of adaptive routing. **Note.** Panel A summarizes the node-wise procedure used to grow the conditional-inference tree. At each node, the algorithm tests the global null hypothesis of independence between the predictors and the outcome(s). If the null hypothesis is rejected, the predictor with the smallest adjusted *p* value is selected, and the admissible binary split that maximizes the test statistic is chosen; otherwise, the node becomes terminal. Panel B provides a schematic illustration of the resulting adaptive routing structure. The dark nodes and terminal node connected by dashed lines indicate one possible branch of the tree. Starting from the root node, all respondents receive the same initial item; subsequent items depend on the responses provided at each preceding node. This recursive process continues until a terminal node (TN) is reached. Each TN corresponds to a subgroup of respondents defined by a specific response pattern and stores the predicted score(s) associated with that branch.

*CAT development:* A real-data simulation approach with cross-validation was used to develop the CAT. Specifically, a multivariate conditional-inference tree (ctree) was estimated on the training dataset (67%; random split). In the model the 15 items of the HRI-15 [11,44] were used as predictors, while the HRI-15 total score (sum) and the five standardized (*M* = 0, *SD* = 1) scores on its subscales (anthropophobia, agoraphobia, paranoia, lethargy, depressive mood) were the joint outcome variables. The ctree algorithm, as with other regression trees, employs recursive binary partitioning [102]. However, in this framework, in contrast to conventional approaches, both variable and splitting rule selection are determined by permutation tests. This allows for the management of multiple outcomes within a single tree and reduces selection bias [103–105]. At each node, the algorithm tests a global null hypothesis of independence between the predictors and the outcome variable(s); if the null hypothesis is not rejected, the node becomes a leaf. If the null hypothesis is rejected, the predictor with the smallest *p*-value (adjusted for multiplicity) is selected for splitting; and for that predictor, all possible binary partitions are evaluated, choosing the split that maximizes the test statistic across branches. For a single continuous outcome, the split statistic can be interpreted as a standardized difference between the mean outcome values in the two child nodes defined by a candidate split. For ordered outcomes, the comparison is instead based on a linear-rank statistic computed from the outcome scores/ranks. For multivariate outcomes, the statistic combines the differences between the two child nodes across the outcome vector into a single quadratic-form test statistic [11,44]. At each split, specific node-size constraints have to be met. Specifically, the constraints pertain to the minimum number of observations required in the nodes (or the sum of the case weights) to attempt a split (i.e., the minimum split or “*minsplit*”) and the minimum number of observations that must be included in each leaf (i.e., the minimum bucket or “*minbucket*”). The thresholds for these parameters are set to balance tree parsimony against predictive accuracy (higher thresholds favor simplicity; lower thresholds allow for a closer fit, at the risk of overfitting). At the end of the branches growing process, leaves provide piecewise-constant predictions representing the mean of the outcome variable(s) in a specific subset (which is equivalent to the intercept of an intercept-only model for that subset). The branches growth stops when no covariate shows sufficient association with the outcome(s) at the prespecified significance threshold (i.e., “*mincriterion*”). This threshold is the *p*-value that determines whether a node should be split (it is typically set as 1 − α; e.g., a *mincriterion* of .95 corresponds to α .05, meaning that *p* ≤ 0.05 is required for a split) or when node/leaf sample-size constraints (i.e., *minsplit*/*minbucket*) are not met; therefore, no pruning or smoothing is required. In the present study, candidate tuning configurations were examined over a broad grid of plausible values for *mincriterion* (.10, .20, .50, .70, .90), *minsplit* (10, 20, 50), and *minbucket* (10, 20, 50). Candidate configurations were first compared by 5-fold cross-validation within the training subset. They were then refitted on the full training subset and evaluated on the corresponding independent test subset to examine out-of-sample performance and the sensitivity of agreement, prediction error, and item savings to alternative hyperparameter settings. The final configuration was chosen to balance predictive accuracy and administration efficiency. Analyses were performed in the statistical environment R [91] using the partykit package [104,106,107] (Fig 1).

***Evaluation of CAT performance:*** Once the tree structure had been defined using the above-described procedure on the training dataset, it was used to instruct an adaptive assessment procedure based on respondents’ answers in the test dataset, for which complete responses were available (i.e., real data simulation). The procedure simulated the administration to participants of only the items that corresponded to the branch they were moving along based on their responses, and it produced total (sum score) and subscale (*z*-score) score estimates. On this dataset (not used to develop the tree), the CAT performance was evaluated in terms of efficiency and accuracy. The efficiency of the CAT procedure relative to the full-length test was measured by item savings (the average number of items administered and the corresponding reduction percentage) and evaluated using *t*-tests. Accuracy, interpreted as score reproduction, was quantified using Pearson’s correlation coefficients (*r*) and mean absolute error (MAE). Correlations indicate the degree to which CAT scores align with those obtained by administering all items (a higher *r* indicates better performance), while MAE captures the average absolute deviation between CAT and full-length scores (a lower MAE indicates closer alignment). Paired samples *t*-tests were also run to compare CAT-derived scores with full-length scores. The *t*-tests assess the null hypothesis that the mean of CAT scores does not differ from that of full-length scores.Agreement between CAT- and full-length scores was assessed also using intraclass correlation coefficients computed on the independent test set for each HRI-15 scale and the total score, with a two-way model, absolute agreement, single measure (ICC(A,1)). Interpretation followed [108]: ICC < .50 poor, .50–.75 moderate, .75–.90 good, > .90 excellent.

The performance of the CAT was also evaluated in terms of its accuracy in classifying risk. The cut-off score derived from the ROC curve estimated on the training data set was applied to both the total scores obtained through the CAT and the total scores obtained from the full-length administration. For both sets of scores, the following metrics were checked: sensitivity, specificity, overall accuracy, and Cohen’s κ coefficients. Cohen’s κ represent chance-corrected agreement between each method’s classifications and the criterion variable (e.g., external variable indicating the tendency to social withdrawn). Higher values indicate that the method’s predicted labels better reflect the actual condition. For the purpose of interpretation, conventional criteria were employed: 0–0.20 mild, 0.21–0.40 fair, 0.41–0.60 moderate, 0.61–0.80 substantial, and ≥ 0.81 almost perfect agreement [109]; see also [71]). To directly compare the two methods, we used McNemar’s χ² test (*df* = 1) on the paired classifications relative to the criterion variable. A *p*-value less than .05 indicates different error rates between CAT and full-length scores.

Finally, a Latent Transition Analysis (LTA) was conducted to examine the coherence of latent class assignment between the profiles estimated from the full-length and CAT versions of the HRI-15. LTA is a multi-occasion extension of LPA that enables the evaluation of latent profiles across different occasions and the estimation of transition probabilities between them [95]. In this work, the two considered conceptual “occasions” were the two set of scores (i.e., full-length and CAT scores). In the analysis the indicators were the standardized scores on the five HRI-15 dimensions. First, a configural model was specified, holding the structure and number of profiles constant across the two sets of scores, while allowing means and variances of the indicators to be freely estimated by profile and set of scores. This specification permits level differences between full-length and CAT scores while preserving the conceptual alignment of profiles across occasions [95]. Estimation was performed in Mplus 7.4 [56] using the robust maximum likelihood estimator (MLR; [101]) within a mixture-model framework, with time-2 classes regressed on time-1 classes to obtain the transition matrix (row-standardized transition probabilities from the full-length–based profile to the CAT-based profile). Entropy was used as a classification-quality index (values close to 1 indicate more precise classification), as in the preceding LPA. Concordance between the full-length and CAT solutions at the profile level was also considered. Finally, particular attention was paid to the profile transition probabilities across score type (full-length vs. CAT); low off-diagonal probabilities (i.e., high diagonal stability) were taken as evidence that the CAT procedure yields categorizations comparable to the full-length administration.

***Application for adaptive assessment and automatic reporting:*** A Shiny application [91,110,111] was developed for the adaptive administration of the HRI-15 [11], based on the tree structure derived in the training dataset. Computerized administration follows a deterministic traversal of a pre-populated decision tree. At runtime, two external tables are loaded: (i) a tree sheet that lists the internal nodes, the splitting rules, and the outputs of the terminal leaves (i.e., sum score, subscale *z*-score, and LPA derived profile); and (ii) a dictionary that maps item codes to the item text and scale to which they belong.

Administration begins at the root (the first non-leaf node not referenced as a child). At each step, the engine presents the item indicated by the current node’s variable, records the response, evaluates the split, and advances to the next item based on the response and tree structure. If a subsequent node requests a variable that has already been answered, the engine automatically advances using the stored response until a new variable is requested or a terminal leaf is reached. The stopping rule is the first terminal leaf encountered. The application then returns leaf-specific predictions: the HRI-15 total score, the *z*-scores of the five subscales, and the latent class. The results are immediately displayed in a concise, profile-based report. The total score is interpreted based on the ROC-derived decision rule (cutoff ≥ 42), and the dimensional profile is graphically represented to support a two-step workflow in the applied settings: threshold-based triage followed by profile-driven personalization. The system is implemented in Shiny and relies on external files (Excel) for both the tree structure and the dictionary, allowing content to be updated without code changes. Main dependencies include shiny, readxl, dplyr, stringr, and ggplot2, along with custom functions for parsing and matching of path rules. The interface presents standardized instructions and, for each item, and a short guide to interpret results.

## Results

### Receiver operating characteristic (ROC) analysis

**Fig 2:**
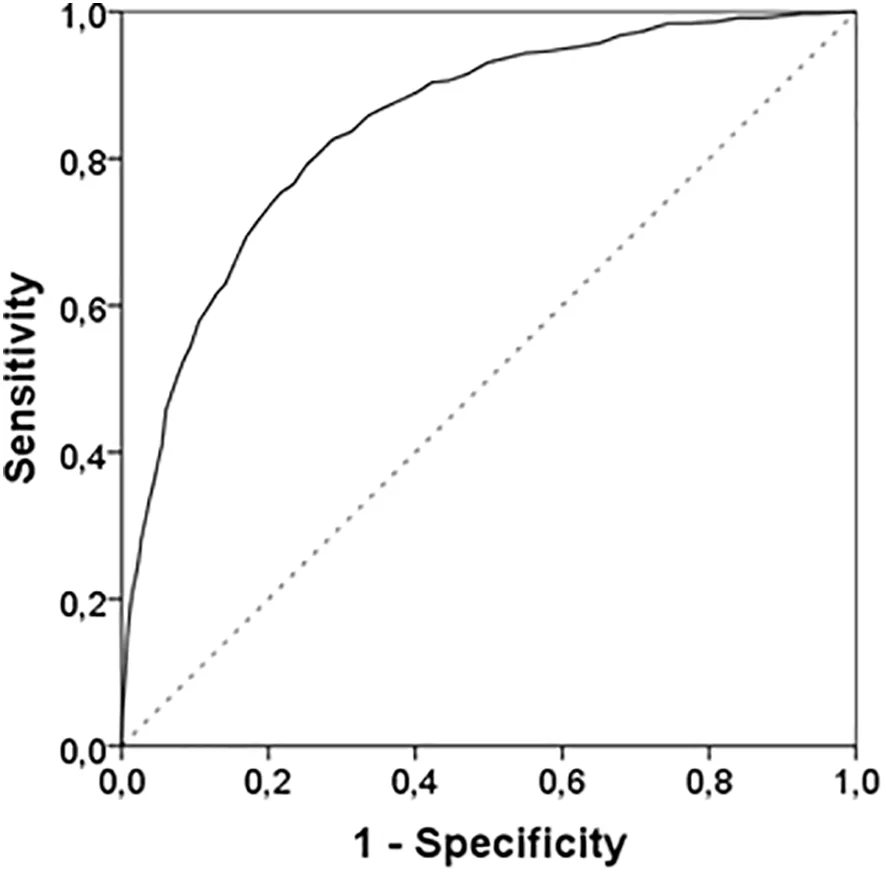
ROC on Training dataset (*N* = 5,866). Note. Area under the curve was .846.

ROC analysis was conducted on the training dataset in order to identify the optimal cut-off score for the instrument. This analysis was then validated on the testing dataset, with the HRI-15 total score serving as the dependent variable and the item on the tendency to self-confine over the past six months as the external criterion (recoded as *positive* for responses 4–5: “Every week (but not every day)”/ “Every day”). According to this criterion, there were 374 positive cases in the training dataset and 184 in the testing dataset. In the training data set, the area under the ROC curve (AUC) was .846, CI [.826, .865] (Fig 2). The Youden index indicated an optimal cut-off score of ≥ 42. The performance metrics achieved using this cut-off score were .751, .791, and .748 for accuracy, sensitivity, and specificity, respectively. The corresponding 95% confidence intervals were [.740, .762] for accuracy, [.747, .832] for sensitivity, and [.737, .760] for specificity. When the same cut-off score was applied to the testing dataset, the corresponding values were AUC = .837, 95% CI [.809, .865], accuracy = .726, 95% CI [.710, .742], sensitivity = .783, 95% CI [.716, .840], and specificity = .722, 95% CI [.705, .739] (further details are reported in S1 and S2 Tables in the S1 File). In summary, a cut-off score of ≥ 42 showed acceptable ability to identify individuals at risk.

A sensitivity analysis using a stricter criterion definition, in which only the response “Every day” was coded as positive, yielded the same optimal integer cut-off score (≥ 42). In the training dataset, the AUC was .819, 95% CI [.780, .858], with accuracy = .725, 95% CI [.713, .736], sensitivity = .771, 95% CI [.685, .843], and specificity = .724, 95% CI [.712, .735]. In the testing dataset, the corresponding values were AUC = .794, 95% CI [.731, .858], accuracy = .700, 95% CI [.683, .717], sensitivity = .764, 95% CI [.630, .868], and specificity = .699, 95% CI [.682, .716]. Overall, the convergence of the primary and sensitivity analyses supported the robustness of the ≥ 42 threshold.

Supplementary calibration analyses based on logistic regression models estimated in the training dataset indicated acceptable agreement between predicted and observed risk probabilities in the testing dataset. In the primary analysis, the Brier score was 0.052, the calibration intercept was −0.087, and the calibration slope was 0.941, indicating only modest deviations from ideal calibration (see Supplementary Tables S3–S5 Tables and S6 Fig in S1 File).

### Latent profile analysis

A total of five models, with one to five classes, were estimated. The results were evaluated using a combination of statistical and qualitative criteria (Table 1). The information criteria (i.e., AIC, BIC and SABIC) decreased sharply up to four classes, whereas the improvement from four to five classes was more modest. Entropy was highest for the four-class solution (0.877), indicating clearer classification. The VLMR/LMR tests were significant at the transitions from 2 to 3, 3–4, and 4–5 classes (*p* < 0.01), indicating continued improvement in fit with increasing model complexity. However, the incremental improvement from four to five classes was comparatively limited, whereas the four-class model yielded the clearest classification and an interpretable configuration with well-defined class proportions (52.4%, 17.5%, 15.0%, and 15.2%). The four-profile solution was therefore retained as the preferred solution, as it offered the best balance among fit improvement, interpretability, parsimony, and classification quality. Additional classification diagnostics for the retained solution, including average posterior probabilities and class-specific parameter estimates, are reported in (S7–S10 Tables in S1 File). Supplementary robustness analyses conducted in the training and testing subsets also supported the stability of the four-profile configuration (S11 Table and S12 Fig in S1 File).

**Table 1:** Fit indices for latent profile analysis.

Class	1	2	3	4	5
Free parameters	10	16	22	28	34
Loglikelihood	−62111.530	−53576.928	−51740.522	−50211.228	−49772.389
Scaling	0.830	1.471	1.534	1.664	1.751
AIC	124243.060	107185.857	103525.043	100478.457	99612.778
BIC	124313.834	107299.095	103680.746	100676.623	99853.409
SABIC	124282.056	107248.250	103610.834	100587.644	99745.363
Entropy		0.891	0.837	0.877	0.867
Vuong-Lo-Mendell-Rubin		17069.20	3672.81	3058.59	877.68
*p*		0.000	0.000	0.000	0.005
Lo-Mendell-Rubin Adjusted LRT		16761.452	3606.594	3003.442	861.855
*p*		0.000	0.000	0.000	0.006
Class size					
		2798	4629	4587	1264
		5957	2627	1532	4375
			1499	1309	1332
				1327	980
					804

**Fig 3:**
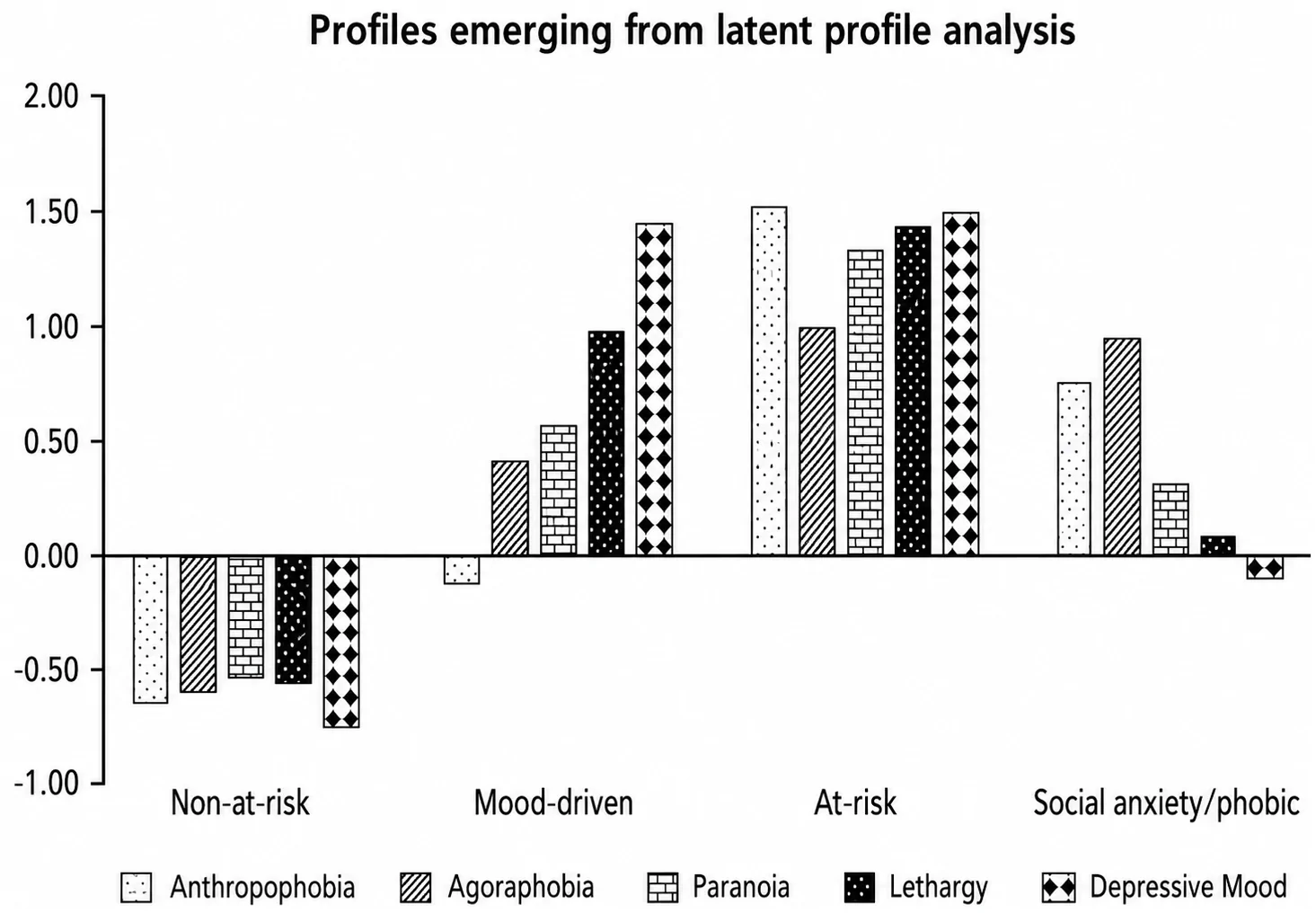
Profiles emerging from latent profile analysis. *Note*. Bars represent standardized scores on Anthropophobia, Agoraphobia, Paranoia, Lethargy, and Depressive Mood for the profiles identified via latent profile analysis (LPA). Positive values indicate scores above the sample means; negative values indicate scores below the sample means. The four profiles are: (1) Non-at-risk, (2) Mood-driven, (3) At-risk, and (4) Social anxiety/phobic. Scores are standardized within-sample (*M* = 0, *SD* = 1). Within-class standard deviations were 0.585 for Anthropophobia, 0.547 for Agoraphobia, 0.806 for Paranoia, 0.702 for Lethargy, and 0.499 for Depressive Mood; these values were constrained to be equal across classes in the retained model.

Fig 3 illustrates the profiles derived from the four-class solution, which clearly show distinct profiles. The first group, which is by far the largest, exhibits sub-mean levels across all dimensions, indicating the absence of clinically significant signs of social withdrawal. The second group is primarily characterized by a depressed mood and low energy levels. Social avoidance (agoraphobia and anthropophobia) is a secondary characteristic, while suspicion or distrust is minimally present. This pattern can be regarded as a form of “secondary” withdrawal, where social disengagement is a consequence of pervasive sadness and reduced activity. The third profile most closely aligns with the true hikikomori pattern, which is characterized by social avoidance alongside symptoms such as paranoia, lethargy, and depressed mood, indicating a more pronounced withdrawal syndrome. The fourth group is characterized by anxiety-driven withdrawal, where the main challenges involve social interactions and environmental contexts (agoraphobia and anthropophobia), while depressed mood and low energy levels are comparatively less pronounced. In essence, the identified groups appear to distinguish between individuals without clinical indicators (Class 1, “non-at-risk”); individuals whose withdrawal is predominantly mood-driven (Class 2, “Depressive–lethargic”); individuals exhibiting a characteristic hikikomori pattern (Class 3, “at-risk”); and individuals whose withdrawal is primarily influenced by phobic and social anxiety traits (Class 4, “Social-anxiety/phobic”).

**Fig 4:**
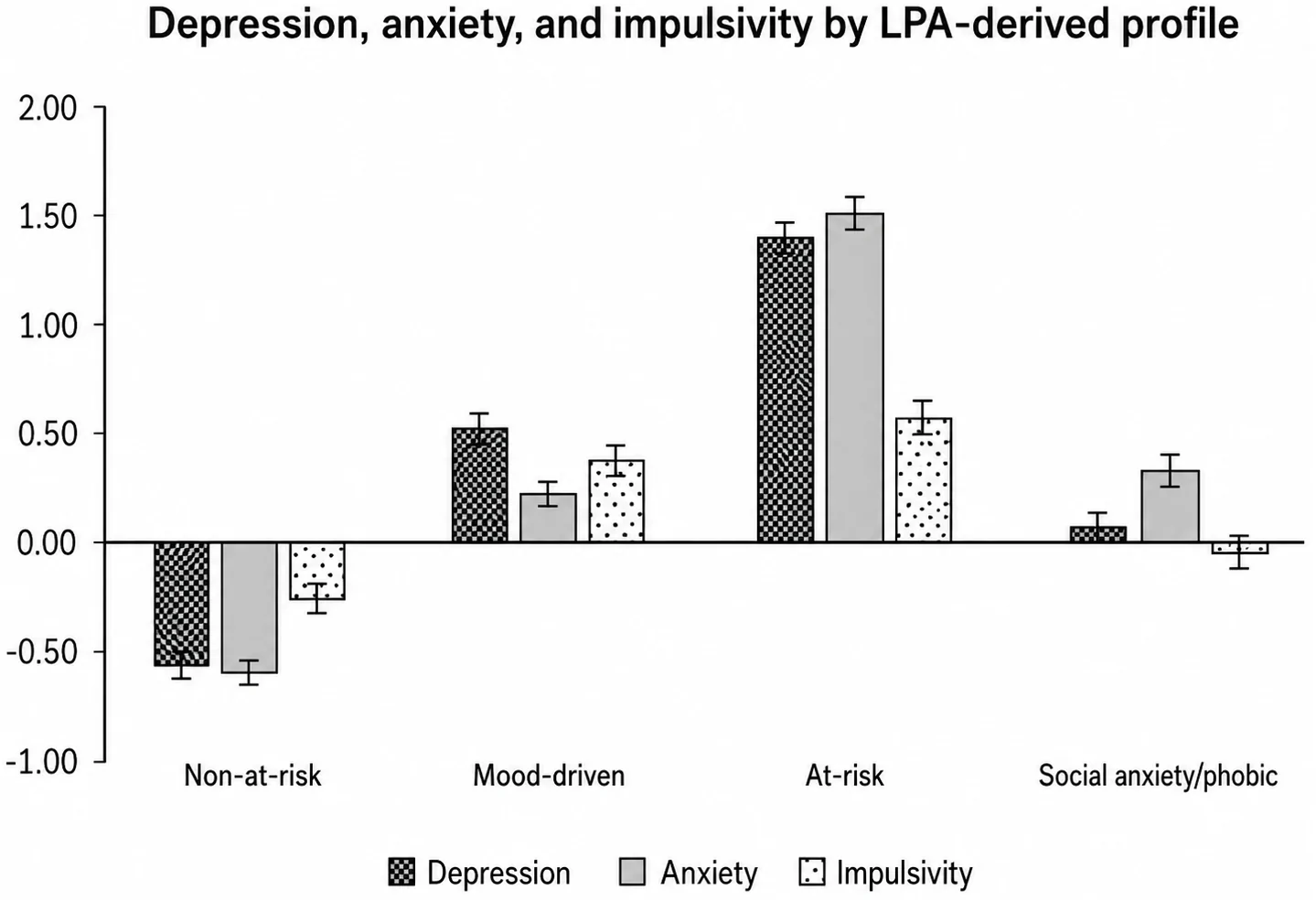
Depression, anxiety, and impulsivity by LPA-derived profile. *Note*. Bars represent standardized (z) scores for depression, anxiety, and trait impulsivity across the four profiles identified by latent profile analysis (LPA). Higher values indicate greater symptom severity/impulsivity. Scores are standardized within-sample (*M* = 0, *SD* = 1). Error bars show 95% confidence intervals around the mean; substantial overlap suggests non-significant differences. Post hoc pairwise comparisons indicated that all between-class differences were statistically significant (*p* < .01).

Differences between profiles extended systematically to the other psychological variables and risk behaviors considered. ANOVAs revealed large, significant differences for depressive symptoms (*F*(3, 2742) = 2496.7, *p* < .001, *η*² = .518) and social anxiety (*F*(3, 2688) = 2665.6, *p* < .001, *η*² = .545), as well as medium differences for impulsivity (*F*(3, 3055) = 301.4, *p* < .001, *η*² = .103). As shown in Fig 3, Class 1 (i.e., the non-at-risk profile) displays below-average levels of social anxiety, depression and impulsivity. Class 2 (i.e., the mood profile) shows slightly above-average anxiety and impulsivity, and moderately elevated depression. Class 3 (i.e., the at-risk profile) combines high anxiety and depression with moderately high impulsivity, constituting the highest-risk profile. Class 4 (i.e., the social anxiety profile) is characterized by above-average social anxiety. Overall, this pattern aligns with the expected profile for each class (Fig 4).

Notable differences across profiles were also observed concerning risky behaviors and habits (Tables 2 and 3). For instance, daily engagement with social media and video games use varied systematically according to the hikikomori risk profile. Regarding gaming, the χ^2^ test yielded a significant result (χ² = 145, *p* < .001, *V* = .074), indicating a small effect size. For social media use, the association was more pronounced (χ² = 580, *p* < .001, *V* = .149), indicating a small to medium effect size. Examination of standardized residuals (|*z*| > 1.96) indicated that profiles characterized by depressed mood and those defined as “at-risk” (Classes 2–3) were more likely to report high daily social media engagement (from ≥ 3 to > 9 hours/day), whereas “non-at-risk” and “social anxiety” profiles (Classes 1 and 4) more frequently reported low levels of use. With respect to gaming, very intensive engagement (7–8 hours/day) was more likely in Class 3.

**Table 2:** Associations between LPA-derived profiles and behavioral indicators.

Class	Variable	*N*		χ^2^	*V*
		Yes	No		
1	Social Challenge involvement	259	4328	9.92*	0.0337
2		118*	1414		
3		72	1237		
4		88	1239		
1	Drinking alchool	2130***	2457***	321***	0.192
2		1064***	468***		
3		829***	480***		
4		623**	704**		
1	Cannabis use	489	1840	77.1***	0.123
2		338***	756**		
3		211	**735**		
4		109***	654*		
1	Sexting	391***	1938**	81.1***	0.126
2		303***	791*		
3		265***	681*		
4		148	615		
1	Seeking support	3154***	1433***	1119***	0.357
2		573***	959***		
3		292***	1017***		
4		629**	698**		

*Note.* Observed counts (Yes/No) for each behavior are shown by profile: (1) Non-at-risk, (2) Mood-driven, (3) At-risk, and (4) Social anxiety/phobic. The χ² statistic (*df* = 3 for all tests) evaluates the association between profile membership and each variable; Cramér’s *V* indexes effect size. Asterisks on cell counts flag standardized residuals whose absolute value exceeds conventional thresholds. Boldface indicates positive standardized residuals (over-representation); significant negative residuals (under-representation) are not bolded. *p < .05, **p < .01, ***p < .001.

**Table 3:** Associations between LPA-derived profiles and daily video-game and social-media use.

Class	Variable	*N*					χ^2^	*V*
		Less than 1 hour	1–2 hours	3–6 hours	7–8 hours	9 hours or more		
1	Videogames use	2052***	1519***	803*	128	85	145***	0.074
2		868***	350***	237	40	37		
3		746***	304***	179*	51*	29		
4		727*	362	185	30	23		
1	Social media use	1367***	1684**	1159***	228***	149***	580***	0.149
2		190***	518	581***	143***	100***		
3		135***	352***	561***	159***	102***		
4		347*	461	398	76	45		

*Note.* Observed counts by usage category (Less than 1 hour, 1–2 hours, 3–6 hours, 7–8 hours, 9 hours or more) are shown for each latent profile: (1) Non-at-risk, (2) Mood-driven, (3) At-risk, and (4) Social anxiety/phobic. For each variable (video-game use; social-media use), the χ² test assesses independence between usage category and profile membership (*df* = 12 for both tables); Cramér’s *V* indexes effect size. Asterisks on cell counts flag significant deviations based on standardized residuals. Boldface indicates positive standardized residuals (over-representation); significant negative residuals (under-representation) are not bolded. *p < .05, **p < .01, ***p < .001-

Participation in social media challenges was weakly but significantly associated with class membership (χ²(3) = 9.92, *p* < .05, *V* = .034). Standardized residuals indicated greater participation among individuals categorized on the “depressive” profile (Class 2). Regarding sexting, assessed only in the high-school subsample, small but statistically significant differences emerged across classes (*χ*²(3) = 81.10, *p* < .001, *V* = .126), with the “non-at-risk” group (Class 1) showing a lower propensity to send erotic/sexual content, and the depressive and “at-risk” groups (Classes 2 and 3) showing a higher propensity.

With respect to substance use, a small-to-moderate association was found between alcohol consumption and class membership (*χ*²(3) = 321, *p* < .001, *V* = .192), whereas cannabis use demonstrated a significant but small association (*χ*²(3) = 77.10, *p* < .001, *V* = .123). Standardized residuals indicated that the probability of alcohol use was higher in classes characterized by depressive mood and in the “at-risk” group (Classes 2 and 3) but lower in the “non-risk” and “anxious” profiles (Classes 1 and 4). Cannabis use was more likely in the depressive class (Class 2) and less likely in the anxious-phobic class (Class 4).

Finally, the protective tendency to seek support by talking with parents or caregivers differed markedly across profiles (*χ*²(3) = 1119, *p* < .001, *V* = .357). Standardized residuals indicated that Classes 2, 3, and 4 were more inclined to speak with reference figures, whereas only the “non-at-risk” profile (Class 1) was less inclined to do so.

It is interesting to note that the profile membership from LPA was associated with the risk status both relative to the criterion variable (χ²(3) = 284, *p* < .001, Cramér’s *V* = .313) and to the dichotomous classification derived from the ROC cut-off score (χ²(3) = 1833, *p* < .001, Cramér’s *V* = .796). The pattern was consistent in the testing dataset and in the total sample. In the testing dataset, standardized residuals (|*z*| > 1.96) indicated that Class 3 (i.e., “at-risk” profile) was more likely to be positive and to be classified as positive (*p*s < .001), whereas Class 1 (i.e., “non-at-risk” profile) was more likely to be negative and to be classified as negative (*p*s < .001). Classes 2 and 4 (i.e., “depressive” and “anxious” profiles) were more likely to be classified as at-risk (*p*s < .001; in the total sample Class 2 also showed a higher likelihood of being positive according to the criterion, *p* < .001) (Table 4).

**Table 4:** Associations of LPA-derived profiles with the external Criterion and ROC-based classification.

	External Criterion				ROC-based classification			
	*N*				*N*			
Class	Negative	Positive	χ^2^	*V*	Negative	Positive	χ^2^	*V*
1	1470*	16***	284***	0.313	1486***	0***	1833***	0.796
2	462	39			308*	193**		
3	364***	107***			0***	471***		
4	409	22			200***	231***		

*Note*. Observed counts (Negative/Positive) are shown for each profile under two dichotomies: External Criterion (left; “self-confinement every week or every day” = Positive) and ROC-based classification (right; HRI-15 total score cut-off ≥ 42 = Positive). For each dichotomy, the overall χ² test (*df* = 3) evaluates the association between profile membership (1 - Non-at-risk, 2 – Mood-driven, 3 – At-risk, and 4 – Social anxiety/phobic) and risk status; Cramér’s *V* indexes effect size. Asterisks on cell counts mark significant cellwise deviations based on standardized residuals. Boldface indicates positive standardized residuals (over-representation); significant negative residuals (under-representation) are not bolded. *N* = 2,889 (testing dataset).*p < .05, **p < .01, ***p < .001.

### CAT performance

The ctree algorithm was tuned on the training data set by examining a grid of plausible hyperparameter settings for *mincriterion*, *minsplit*, and *minbucket*, taking into account 5-fold cross-validated performance together with item savings and tree complexity. The cross-validation and sensitivity analyses indicated that the configuration *mincriterion* = .10, *minsplit* = 10, and *minbucket* = 10 provided a balanced compromise between predictive accuracy and administration efficiency (detailed tuning and sensitivity results are reported in Tables S13 to S16 in the S1 File).

When refitted on the full training data set with the selected hyperparameter configuration, the ctree algorithm yielded a tree structure with 349 leaves, with item 15 (“*I feel a sense of inner emptiness*”) at the root. Leaves stored predictions for the HRI-15 total score and the standardized scores on the five subscales; branch depth ranged from 3 to 14 nodes.

This structure was then applied to the independent testing set to drive adaptive administration. The number of administered items ranged from 3 to 14, with an average of 7.03 items, corresponding to a 53.13% saving relative to the full-length version (15 items). A *t*-test on the number of administered items confirmed that the mean was significantly lower than 15 (*t*(2888) = −143, *p* < .001, *d* = −2.66).

Regarding accuracy, agreement between scores obtained with full administration and those estimated adaptively was high: correlations ranged from .767 to .997, with good to excellent values of ICC (from .751 to .997) and low MAEs (Table 5). In addition, paired-samples *t-*tests indicated no significant differences between full-length and CAT scores on any scale. Moreover, applying the ROC-based cutoff score to the two sets of score yielded comparable classification performance: CAT scores achieved .73 accuracy, with sensitivity .73, specificity .73 and Cohen’s κ .17 (mild), while full-length scores achieved the same overall accuracy (.73) with sensitivity .78, specificity .72, and κ .18 (mild). Consistent with this, McNemar’s test showed no significant difference in error rates when applying the cutoff of ≥ 42 to CAT or full-length scores, χ²(1) = .194, *p* = .66 (further details on administration diagnostics, CAT classification, and score-discrepancy analyses between CAT-derived and full-length scores are reported in Tables S17–S23 in the S1 File).

**Fig 5:**
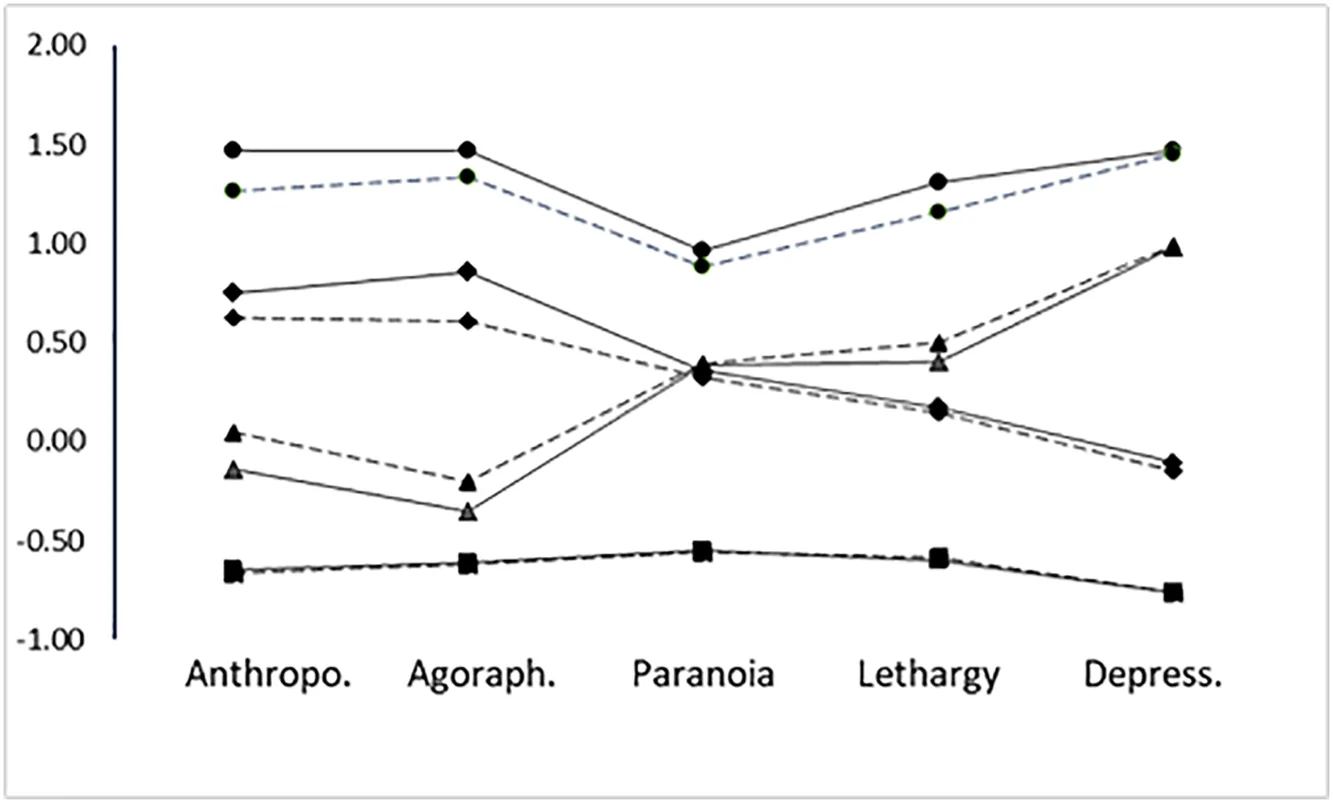
Profiles emerging from LTA based on full-length test and CAT administration (*N* = 2,889; testing dataset). *Note***.** Profile means from full-length test (solid) and CAT administration (dashed) across the five HRI dimensions. Four profiles are shown: (1) Non-at-risk, (2) Depressive–lethargic, (3) At-risk, and (4) Social-anxiety/phobic. Lines are plotted in black with distinct markers per profile.

Further evidence for the comparability of CAT and full-length scores came from a latent transition analysis (LTA). The five HRI-15 dimensions served as indicators at two occasions (full-length scores represent the first occasion; and CAT scores the second). A configural LTA model with four profiles per occasion was estimated. For interpretability, CAT-based classes were remapped to align with full-length-based profile meanings based on class-specific means. The results showed excellent classification quality (entropy = .959; AIC = 53,180.38; BIC = 53,568.35; SABIC = 53,361.82; *N* = 2,889). Profile-level agreement between full-length and CAT profiles was high (diagonal transition probabilities from .927 to .954; *M* = .943), indicating that the CAT yields categorizations comparable to the full-length administration (Table 6). Profile shapes were substantially superimposable across occasions (Fig 5), supporting conceptual alignment.

**Table 5:** Agreement between full-length and CAT scores on the HRI-15: descriptive and comparative statistics (*N* = 2,889; testing dataset).

	MeanFull-length score	MeanCAT score	MAE	*r*	ICC(A,1) [95% CI]	*t*	*p*	Cohen’s *d*
Sum score HRI-15	34.654	34.720	3.980	.925	0.922 [0.92, 0.93]	−.649	.517	−.012
*z* Anthropophobia	.020	.018	.445	.793	0.778 [0.76, 0.79]	.206	.837	.004
*z* Agoraphobia	.004	.018	.421	.811	0.797 [0.78, 0.81]	−1.255	.21	−.023
*z* Paranoia	.013	.009	.466	.772	0.757 [0.74, 0.77]	.387	.699	.007
*z* Lethargy	.017	.028	.465	.767	0.751 [0.74, 0.77]	−.912	.362	−.017
*z* Depressive Mood	.023	.024	.025	.997	0.997 [0.99, 0.99]	−.187	.852	−.003

*Note*. The table reports mean scores from the full-length administration and the CAT, the mean absolute error (MAE; in the metric of each outcome), the Pearson correlation (*r*) between methods, and paired-samples *t*-test results comparing CAT vs. full-length (*df* = 2,888) with Cohen’s *d* for paired differences (positive *d* indicates CAT > full-length). ICC represents intraclass correlation coefficient (ICC(A,1)).

**Table 6:** Latent transition probabilities.

	C2#1	C2#2	C2#3	C2#4
C1#1	.954	.000	.046	.000
C1#2	.000	.937	.000	.063
C1#3	.048	.000	.952	.000
C1#4	.000	.073	.000	.927

*Note.* Rows sum to 1.

### Development of App

The app is provided in the S1 File. It administers the test adaptively and returns results in real time, including: (a) the total score on the general scale, (b) standardized scores for each subdimension, (c) a bar chart displaying the subdimension scores, and (d) an indicator of whether the ROC-based cut-off is exceeded, (e) along with profile membership according to the LPA classification.

**Fig 6:**
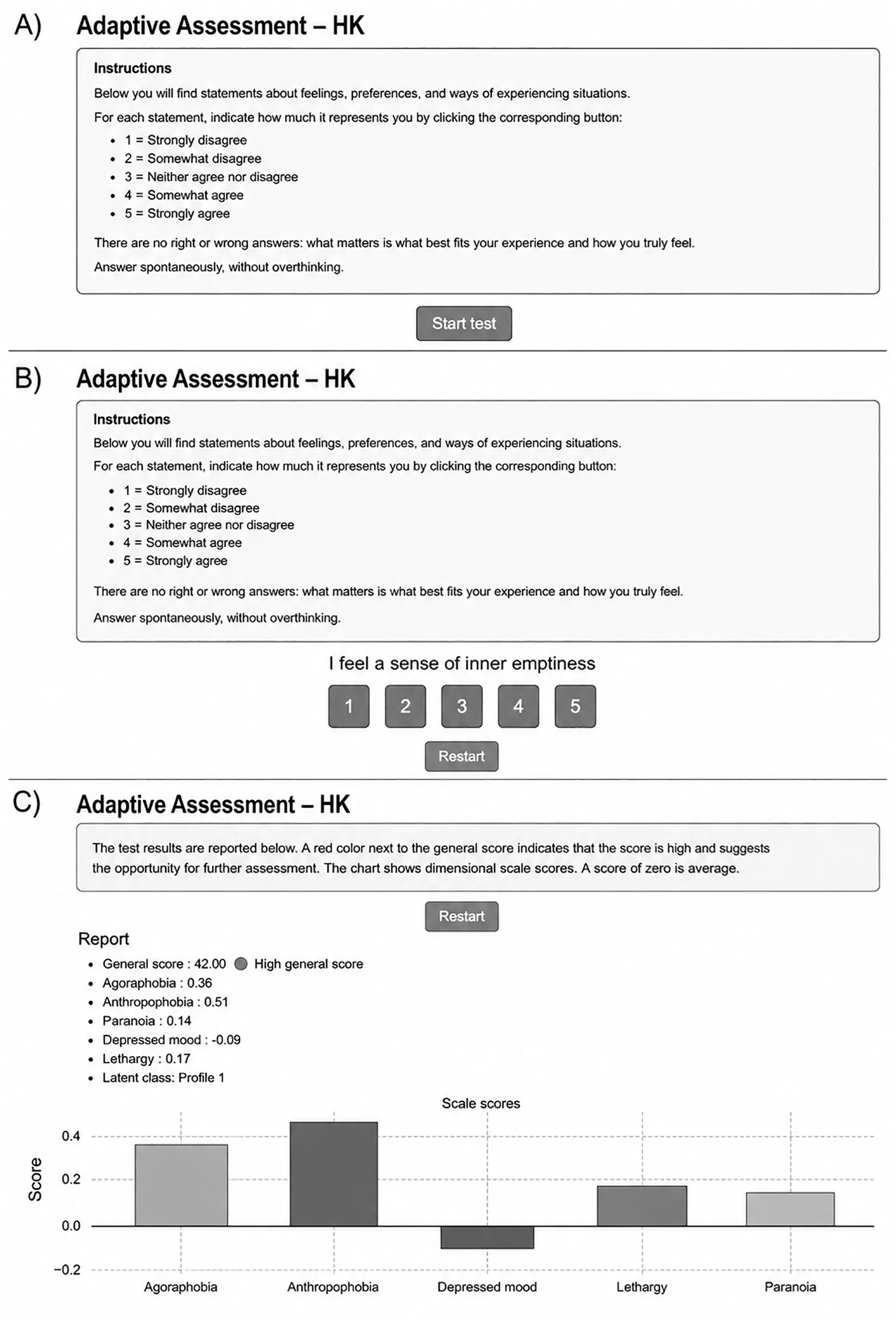
Shiny app for adaptive administration and automatic reporting of the HRI-15. *Note*. Screens from the adaptive assessment application (S1 File). A) Landing page with standardized instructions and “Start test.” B) Item screen showing a single prompt with five response buttons (1–5; “Not true for me” to “Very true for me”). Items are administered adaptively based on prior answers; instructions remain visible and a “Restart” option is available. C) Results screen displaying the total score, standardized subscale scores (z), LPA-based profile assignment, a ROC–cutoff risk flag, and a bar chart of subscale scores. Subscale scores are standardized within-sample (*M* = 0, *SD* = 1). The risk indicator reflects the ROC-derived cutoff (≥ 42). Color coding of the flag is not visible in grayscale of this screen.

The home screen presents instructions; users proceed by tapping “Start test” (Image A, Fig 6). This initiates item administration: respondents select an option, which routes them to the subsequent item (Image B, Fig 6). Instructions remain visible on the page, and a button is available to restart the assessment if needed.

Upon completion, a results screen displays the scores, guidance for interpretation, and a bar chart of dimensional scores (Image C, Fig 6).

## Discussion

Originally described in Japan, the “hikikomori” phenomenon is now recognized as a relevant issue across cultural boundaries, spreading also in the USA and EU [3,4,7,8,10–14]. The available estimates suggest a prevalence of approximately 1.2% and a marked involvement among younger age groups, with a typical onset in adolescence [17,20,24,35,36]. Given its prevalence and the long-term repercussions on personal development, educational (occupational) trajectories, and mental health, the implementation of early identification and large-scale screening strategies have become imperative [1,16,19,37–41].

To address these challenges, this study aimed to refine the diagnostic categorization of hikikomori risk and to develop and validate an ML-based CAT for its assessment. The HRI-15 was selected as the target instrument because it is brief, developed through international collaboration, psychometrically sound, and multidimensional [11,44]. In particular, the five dimensions of the scale are derived from psychometric studies designed to measure hikikomori symptomatology and its correlates, as theorized by Saitō (1998) [8,44]. These dimensions provide a detailed description of the phenomenon, allowing the instrument to capture all the key aspects of the construct. However, this richness has not yet been fully exploited in applied contexts.

To achieve the research objectives, the study examined a representative national sample of Italian adolescents (*N* = 8,755) and employed a combination of methodologies: ROC analysis to determine an optimal cut-off on the HRI-15 total score, LPA to delineate multidimensional risk profiles, and ML algorithms to develop the adaptive test.

Concerning ROC analyses, the findings indicated a threshold of ≥ 42 as the optimal cut-off score. This threshold showed adequate discriminative ability (AUC = .85) and an acceptable balance between sensitivity and specificity (from .72 to .79). It is important to note that this threshold is higher than that suggested by previous studies (≥ 37; AUC = .80; [11]). However, the current estimate may be considered more robust, as it was obtained through cross-validation in a larger sample and by using a more detailed external criterion based on the frequency of social withdrawal. In addition, the threshold proved to be stable across sensitivity analyses and showed overall acceptable calibration. Nevertheless, this criterion should be interpreted with appropriate caution. Although it captures a central behavioral manifestation of hikikomori and was therefore suitable for the study’s screening purpose, it was based on a single self-report item rather than on an independent clinical assessment. Consequently, the identified threshold should be evaluated as a pragmatic screening indicator rather than as a definitive diagnostic benchmark. Future research should extend this validation using independent clinical assessments and additional external correlates of functional impairment.

The good performance obtained with ROC analyses suggest that the HRI-15 total score works well in real-world settings as an initial screen. Despite this, risk is not monolithic and depending on a single cut-off may result in the flattening of clinically meaningful heterogeneity, thereby obscuring distinct symptom constellations that could have different implications for support and prevention.

LPA is a person-centered approach that involves the classification of individuals into latent subgroups based on their indicator response patterns, thereby providing a holistic assessment of individual differences that can provide guidance in applied settings [95]. The analysis revealed the presence of four distinct profiles: (1) the non-at-risk group, with below-average levels on all dimensions; (2) the depressive–lethargic group, characterized primarily by depressed mood and low energy, where social avoidance may be secondary and consequent to low mood/energy; (3) the at-risk (hikikomori type) group, with over-average levels on all dimensions (agoraphobia, anthropophobia, paranoia, lethargy, depressed mood); and (4) the social anxiety/phobic group, distinguished by its predominant symptoms of anthropophobia and agoraphobia coupled by smaller elevations in mood/lethargy, a profile that tends to isolate due to difficulties in managing specific situations and interactions. Importantly, these profiles emerged in a sufficiently clear and reproducible manner, as also supported by the supplementary classification diagnostics and split-sample robustness analyses. Overall, these findings make optimal use of the instrument’s multidimensional design, revealing distinct profiles with clear and consistent clinical significance that provide a solid foundation for the development of targeted treatment programs. The validity and clinical utility of the profiles were further enhanced and substantiated by systematic group differences in relation to a series of external variables. In particular, differences emerged in anxiety, depression and impulsivity, with the at-risk profile demonstrating elevated anxiety and depression; the depressive–lethargic profile exhibiting moderate depression and slightly above-average anxiety and impulsivity; and the social anxiety/phobic profile primarily displaying high social anxiety. It is also notable that, as theorized, impulsivity was relatively low across all profiles, confirming theoretical expectations [44,83–86] and supporting the scale’s validity.

Risk behaviors resulted also to be sensibly differentiated across profiles. For example, depressive and at-risk groups exhibited higher daily social media use, while the at-risk profile showed a greater propensity for intensive gaming. Furthermore, depressive and at-risk profiles were associated with a higher incidence of alcohol use and, to a lesser degree, of cannabis use. Overall, these patterns indicate that similar total scores can reflect different risk profiles with distinct correlates and intervention priorities. It is, however, noteworthy that LPA profile membership was associated with risk status both with respect to the external criterion and to the dichotomous classification derived from the ROC cut-off. The “at-risk” profile was more likely to be positive and classified as positive, whereas the “not-at-risk” profile was more likely to be negative and classified as negative. Finally, the depressive” and “anxious” profiles were more likely to be classified as at risk. In practice, the ROC threshold helps decide whom to flag, whereas the profiles clarify what to prioritize clinically.

A central innovation of this work is the development of a multivariate, ML-based CAT that uses a single conditional inference tree (ctree; [102,105,107]) to predict the HRI-15 total score, the five subscale *z* scores, and LPA profile membership. Simulations using empirical data from the test set demonstrated that the CAT required an average of 7.03 out of 15 items, representing a 53% reduction in items needed to complete the assessment (a significant difference relative to the full-length assessment). Despite this efficiency, the procedure successfully reproduced full-length scores with a high degree of accuracy (*r* = .77–.997; MAE for total score 3.98, and from 0.03 to .47 for subscale). Paired-sample *t* tests indicated that CAT scores did not differ significantly from those obtained with the full-length test. In addition, the application of the ROC-based cut-off score to the CAT scores resulted in an accuracy that was comparable to that achieved through the full-length administration, with similar sensitivity/specificity and no differences in error rates. Beyond reproducing scores, LTA showed substantial alignment at the profile level between solutions derived from the full-length and CAT administrations (entropy = .96; diagonal probabilities = .93–.95), indicating that the adaptive procedure preserves the profile structure that gives the instrument its clinical utility.

An additional contribution of the present work is the development of a Shiny-based application for the adaptive administration of the scale, automated scoring, and reporting. From an implementation perspective, the approach is advantageous because it is fast, transparent, and lightweight since the adaptive flow follows a precompiled branch structure, with minimal runtime processing. Reporting is intentionally simple: a total score easily interpretable through the ROC-based thresholds, subscale results expressed as *z*-scores and visually summarized with bar graphs that improve usability in clinical practice, and automatic indication of the most likely profile for the response pattern. A particular strength of this approach lies in integrating the richness of dimensional measurement with a categorical framework that facilitates practical implementation, yielding a synthesis that is both methodologically current and empirically validated [6,112].

Overall, this work offers four concrete advances: (1) clinically useful classes derived from the HRI-15 dimensions, easily computed and interpreted, with distinct external correlates to guide targeted interventions; (2) a cross-validated threshold (≥ 42) for efficient case identification, derived from a frequency-based external criterion and showing improved discrimination; (3) a feasible, real-time adaptive app that returns the total score, subscales scores, risk flag based on ROC-derived cut-off score, and LPA profile assignment, which minimises burden and errors; (4) a generalizable ML workflow (ctree-based multivariate CAT) that illustrates how tree methods can deliver accurate, interpretable, and scalable assessments, moving beyond binary diagnosis toward continuous scores and profile-based categorisation within a single coherent framework.

In applied settings, the results support a two-step strategy: using the threshold for triage and profiles for personalization. Identification of an at-risk profile should trigger a focused approach. Management priorities should include mood and activation for the depressive–lethargic profile, guided exposure and skills training for the social anxiety/phobia profile, and multimodal support addressing withdrawal, mood, suspiciousness and digital use habits for the at-risk profile. In all cases, monitoring of risk behaviours relevant to the profile (e.g., alcohol, cannabis, gaming) is advised. The app operationalizes this flow by returning, at the end of the assessment, a concise, profile-informed report that can be integrated into school, clinical, or epidemiological pathways.

Several limitations warrant caution. First, although the external criterion for ROC analysis improves upon prior dichotomous items by leveraging frequency, it remains a single self-report indicator; future studies should incorporate multi-informant information and clinical interviews. This may have introduced some degree of criterion misclassification, with possible consequences for sensitivity, specificity, and the location of the optimal cutoff. Future studies should incorporate multi-informant information, clinical interviews, and additional external indicators of functional impairment. Second, although the sample is large, it is exclusively Italian and based on school data. Therefore, further validation using samples from other cultures and clinical populations are strongly advocated. In particular, it should be noted that hikikomori is a culturally influenced phenomenon; consequently, the generalizability of the current results to cross-cultural samples appears to be of central relevance. In this regard, some studies have shown that incorporating additional covariates into adaptive models based on tree structures derived from binary partitioning can improve the performance and personalization of the obtained assessment procedures [34]; consequently, future studies could investigate whether socio-cultural variables, gender or other characteristics can enhance the performance of the methodology. Third, although the CAT reproduced scores and profiles well, further studies should evaluate its performance in real-world adaptive administration (e.g., missingness, device variability) and fairness across key subgroups [11]. Future studies should also examine the longitudinal validity of the CAT by assessing the temporal stability of CAT-derived classifications, its sensitivity to change over time, and its performance when embedded in routine school- or clinic-based screening workflows. Future work should evaluate the utility of more advanced computerized therapy or self-monitoring applications that integrate online therapy with data from wearable devices, enabling targeted and timely support. Early identification of hikikomori risk requires tools that are not only valid but also rapid, interpretable, and scalable. By pairing a cross-validated threshold with clinically meaningful profiles, and delivering both via a ctree-based multivariate CAT integrated into an easy-to-deploy app, this work shows that administration time can be halved while preserving accuracy, maintaining alignment with full-length classifications and profiles, and returning immediately actionable information. The approach clarifies how ML can be leveraged, without sacrificing transparency, to strengthen diagnostic assessment and prevention efforts for prolonged social withdrawal. This is consistent with findings indicating that ML is particularly effective for CAT with respect to efficiency, longitudinal use, informativeness, accuracy, and personalization [34,46,52–54,67,68].

## Supporting information

S1 File Supplementary Materials rev. (DOCX)
