# Supplementary material for: Interpretable machine learning for hikikomori screening: The adaptive HRI-15
Source: PLoS One. 2026 Aug 20;21(8):e0355595. doi: 10.1371/journal.pone.0355595 (PMC13492808; doi:10.1371/journal.pone.0355595)
Supplement: Supplementary file 1 [file pone.0355595.s001.docx]

**Supplementary Materials**

**Interpretable Machine Learning for Hikikomori Screening: The Adaptive HRI-15**

**Supplementary ROC analyses**

Detailed ROC results for the HRI-15 total score are reported below. Tables S1 and S2 present classification results under the primary and stricter criterion definitions, including confusion matrices, discrimination indices, and performance estimates in the training and testing datasets. Tables S3 and S4 report the logistic regression models used to derive predicted probabilities, and Table S5 together with Figure S6 documents calibration performance for the full-length HRI-15 in the testing dataset.

**Table S1. Confusion matrices for the HRI-15 cut-off score (≥ 42) under the primary and stricter criterion definitions**

| Analysis | Dataset | Positive cases | TP | FN | TN | FP | Sensitivity | Specificity | Accuracy | Youden’s J |
| --- | --- | --- | --- | --- | --- | --- | --- | --- | --- | --- |
| Primary criterion | Training | 374 | 296 | 78 | 4110 | 1382 | 0.791 | 0.748 | 0.751 | 0.540 |
| Primary criterion | Testing | 184 | 144 | 40 | 1954 | 751 | 0.783 | 0.722 | 0.726 | 0.505 |
| Stricter criterion | Training | 118 | 91 | 27 | 4161 | 1587 | 0.771 | 0.724 | 0.725 | 0.495 |
| Stricter criterion | Testing | 55 | 42 | 13 | 1981 | 853 | 0.764 | 0.699 | 0.700 | 0.463 |

**Note.** The raw ROC threshold was ≥ 42 in all analyses. TP = true positives; FN = false negatives; TN = true negatives; FP = false positives; Youden’s J = sensitivity + specificity − 1. The primary criterion coded as positive responses 4–5 (“Every week, but not every day” / “Every day”), whereas the stricter criterion coded as positive only the response 5 (“Every day”). The training dataset (*n* = 5,866; 48.9% male; *M*_age_ = 14.00, *SD* = 1.99) was used to estimate the ROC curve and identify the optimal cut-off score. The testing dataset (*n* = 2,889; 49.3% male; *M*_age_ = 14.10, *SD* = 1.96) was used to evaluate the performance of the selected cut-off in an independent sample.

**Table S2. ROC performance of the HRI-15 total score under the primary and stricter criterion definitions**

| Analysis | Dataset | *N* | Positive cases | AUC | 95% CI AUC | Sensitivity (95% CI) | Specificity (95% CI) | Accuracy (95% CI) |
| --- | --- | --- | --- | --- | --- | --- | --- | --- |
| Primary criterion | Training | 5866 | 374 | 0.846 | 0.826–0.865 | 0.791 (0.747–0.832) | 0.748 (0.737–0.760) | 0.751 (0.740–0.762) |
| Primary criterion | Testing | 2889 | 184 | 0.837 | 0.808–0.865 | 0.783 (0.716–0.840) | 0.722 (0.705–0.739) | 0.726 (0.710–0.742) |
| Stricter criterion | Training | 5866 | 118 | 0.819 | 0.780–0.858 | 0.771 (0.685–0.843) | 0.724 (0.712–0.735) | 0.725 (0.713–0.736) |
| Stricter criterion | Testing | 2889 | 55 | 0.794 | 0.731–0.858 | 0.764 (0.630–0.868) | 0.699 (0.682–0.716) | 0.700 (0.683–0.717) |

**Note.** The training dataset (*n* = 5,866; 48.9% male; *M*_age_ = 14.00, *SD* = 1.99) was used to estimate the ROC curve and determine the optimal cut-off score, which was then applied unchanged to the testing dataset (*n* = 2,889; 49.3% male; *M*_age_ = 14.10, *SD* = 1.96). The raw ROC threshold was ≥ 42 in all analyses. AUC = area under the curve; CI = confidence interval.

**Table S3. Logistic regression model fit statistics for predicting criterion positivity from the HRI-15 total score in the training dataset**

| Analysis | N | Positive cases | LogLik (full) | LogLik (null) | LR χ² | *df* | *p* | Cox & Snell *R*² | Nagelkerke *R*² |
| --- | --- | --- | --- | --- | --- | --- | --- | --- | --- |
| Primary criterion | 5866 | 374 | -1070.887 | -1391.315 | 640.856 | 1 | < .001 | 0.103 | 0.274 |
| Stricter criterion | 5866 | 118 | -483.780 | -577.742 | 187.924 | 1 | < .001 | 0.032 | 0.176 |

**Note.** Logistic regression models were estimated in the training dataset (*n* = 5,866; 48.9% male; *M*_age_ = 14.00, *SD* = 1.99) to predict criterion positivity from the HRI-15 total score. LogLik = log-likelihood; LR χ² = likelihood-ratio chi-square. The primary criterion coded as positive responses 4–5, whereas the stricter criterion coded as positive only the response 5.

**Table S4. Logistic regression coefficients for predicting criterion positivity from the HRI-15 total score in the training dataset**

| Analysis | Term | B | SE | *z* | *p* | OR | 95% CI for OR |
| --- | --- | --- | --- | --- | --- | --- | --- |
| Primary criterion | Intercept | -6.806 | 0.225 | -30.236 | < .001 | 0.001 | 0.001–0.002 |
| Primary criterion | HRI-15 total score | 0.096 | 0.004 | 22.240 | < .001 | 1.101 | 1.092–1.111 |
| Stricter criterion | Intercept | -7.605 | 0.366 | -20.804 | < .001 | 0.001 | 0.0002–0.0010 |
| Stricter criterion | HRI-15 total score | 0.086 | 0.007 | 12.679 | < .001 | 1.090 | 1.075–1.104 |

**Note.** Coefficients are from logistic regression models estimated in the training dataset (*n* = 5,866; 48.9% male; *M*_age_ = 14.00, *SD* = 1.99). OR = odds ratio; CI = confidence interval.

**Table S5. Calibration metrics for predicted criterion positivity in the testing dataset**

| Analysis | *N* | Positive cases | Brier score | Calibration intercept | Calibration slope |
| --- | --- | --- | --- | --- | --- |
| Primary criterion | 2889 | 184 | 0.0515 | -0.087 | 0.941 |
| Stricter criterion | 2889 | 55 | 0.0179 | -0.131 | 0.896 |

**Note.** Calibration was evaluated in the testing dataset (*n* = 2,889; 49.3% male; *M*_age_ = 14.10, *SD* = 1.96) by applying the logistic regression models estimated in the training dataset to obtain predicted probabilities. The Brier score summarized the overall discrepancy between predicted probabilities and observed outcomes, with lower values indicating better probabilistic accuracy. Ideal values are 0 for the calibration intercept and 1 for the calibration slope. Negative intercept values indicate a tendency to overestimate risk, whereas slopes below 1 indicate somewhat overly extreme predicted probabilities.

**Figure S6. Calibration plot for full-length HRI-15 scores in the testing dataset**

**Note.** Predicted probabilities were obtained by applying the logistic regression model estimated in the training dataset to the full-length HRI-15 total scores in the testing dataset. Points represent grouped observed and predicted probabilities based on deciles of predicted risk. The diagonal line indicates ideal calibration. Closer correspondence between the calibration curve and the diagonal indicates better agreement between predicted risk and observed outcomes.

**Supplementary latent profile analysis results**

Additional classification diagnostics for the retained four-class solution are reported below. The overall fit statistics for the one- to five-class solutions and the profile means for the retained four-class solution are presented in the main text (Table 1 and Figure 3). Tables S7–S10 provide supplementary information on the retained model, including estimated and modal class counts and proportions (Table S7), average posterior probabilities for most likely class membership (Table S8), class-specific indicator means and standard errors (Table S9), and within-class variance parameters (Table S10).

**Table S7. Estimated and modal class counts and proportions for the retained four-class latent profile solution**

| Class | Label | Estimated count | Estimated proportion | Most likely count | Most likely proportion |
| --- | --- | --- | --- | --- | --- |
| 1 | Non-at-risk | 4560.208 | 0.52087 | 4587 | 0.52393 |
| 2 | Mood-driven / Depressive–lethargic | 1527.726 | 0.17450 | 1532 | 0.17499 |
| 3 | At-risk | 1312.924 | 0.14996 | 1309 | 0.14951 |
| 4 | Social-anxiety/phobic | 1354.143 | 0.15467 | 1327 | 0.15157 |

**Note.** Estimated counts and proportions are model-based values derived from the fitted latent profile model. Most likely counts and proportions are based on modal class assignment.

**Table S8. Average posterior probabilities for most likely class membership in the retained four-class latent profile solution**

| Most likely class | Label | Class 1 | Class 2 | Class 3 | Class 4 |
| --- | --- | --- | --- | --- | --- |
| Class 1 | Non-at-risk | 0.965 | 0.018 | 0.000 | 0.017 |
| Class 2 | Mood-driven/Depressive–lethargic | 0.052 | 0.877 | 0.024 | 0.048 |
| Class 3 | At-risk | 0.000 | 0.022 | 0.941 | 0.037 |
| Class 4 | Social-anxiety/phobic | 0.043 | 0.055 | 0.034 | 0.869 |

**Note.** Rows represent most likely latent class membership, whereas columns represent posterior probabilities of belonging to each latent class. Higher diagonal values indicate clearer classification and better separation among classes.

**Table S9. Class-specific indicator means and standard errors for the retained four-class latent profile solution**

| Indicator | Class 1 Non-at-risk | Class 2 Mood-driven / Depressive–lethargic | Class 3 At-risk | Class 4 Social-anxiety/phobic |
| --- | --- | --- | --- | --- |
| Anthropophobia | -0.644 (0.011) | -0.067 (0.059) | 1.537 (0.026) | 0.752 (0.066) |
| Agoraphobia | -0.623 (0.015) | -0.279 (0.026) | 1.536 (0.044) | 0.923 (0.051) |
| Paranoia | -0.536 (0.014) | 0.433 (0.031) | 0.999 (0.021) | 0.348 (0.028) |
| Lethargy | -0.591 (0.010) | 0.543 (0.045) | 1.348 (0.035) | 0.071 (0.033) |
| Depressive Mood | -0.715 (0.011) | 0.974 (0.029) | 1.455 (0.024) | -0.101 (0.041) |

**Note.** Values are class-specific means, with standard errors in parentheses, for the five standardized HRI-15 dimensions used as latent profile indicators. Indicators were standardized in the full sample (*M* = 0, *SD* = 1). Positive values indicate scores above the sample mean, whereas negative values indicate scores below the sample mean.

**Table S10. Within-class variance parameters for the retained four-class latent profile solution**

| Indicator | Variance | *SD* |
| --- | --- | --- |
| Anthropophobia | 0.342 | 0.585 |
| Agoraphobia | 0.299 | 0.547 |
| Paranoia | 0.649 | 0.806 |
| Lethargy | 0.493 | 0.702 |
| Depressive Mood | 0.249 | 0.499 |

**Note.** Variance parameters are reported for the five HRI-15 dimensions used as latent profile indicators. Standard deviations (*SD*) are shown for ease of interpretation and correspond to the square root of the variance. In the retained model, within-class variances were constrained to be equal across classes.

Additional analyses were conducted to examine the robustness of the retained four-class solution across subsamples. Specifically, the same four-profile model was re-estimated separately in the training and testing subsets used elsewhere in the manuscript. The aim of this supplementary analysis was to assess the reproducibility of the retained configuration across samples, rather than to re-determine the optimal number of classes within each subset. Stability was evaluated by comparing profile shapes, class proportions, entropy, and average posterior probabilities after aligning classes across subsets on the basis of substantive profile content. Table S11 reports the corresponding classification diagnostics, and Figure S12 displays the aligned class-specific means for the two subset-specific solutions.

**Table S11. Split-sample stability of the retained four-class latent profile solution across the training and testing subsets**

| Sample | Entropy | Aligned profile label | Estimated proportion | Most likely proportion | Average posterior probability (diagonal) |
| --- | --- | --- | --- | --- | --- |
| Training | 0.873 | Non-at-risk | 0.52610 | 0.53120 | 0.961 |
|  | 0.873 | Social-anxiety/phobic | 0.15834 | 0.15462 | 0.871 |
|  | 0.873 | At-risk | 0.14161 | 0.14200 | 0.938 |
|  | 0.873 | Mood-driven/Depressive–lethargic | 0.17395 | 0.17218 | 0.876 |
| Testing | 0.885 | Non-at-risk | 0.51141 | 0.51298 | 0.970 |
|  | 0.885 | Social-anxiety/phobic | 0.14784 | 0.14849 | 0.860 |
|  | 0.885 | Mood-driven/Depressive–lethargic | 0.17386 | 0.17134 | 0.896 |
|  | 0.885 | At-risk | 0.16689 | 0.16719 | 0.939 |

**Note.** As a supplementary stability check, the retained four-class solution was re-estimated separately in the training subset (*n* = 5,866) and in the testing subset (*n* = 2,889), that is, the same random split already used for the ROC analyses in the main manuscript. Because latent class numbering is sample-specific, profile labels were aligned across subsets on the basis of the class-specific indicator means and their substantive interpretation. Estimated proportions are model-based class proportions, whereas most likely proportions are based on modal class assignment. The last column reports the diagonal elements of the average posterior probability matrix for most likely class membership; higher values indicate clearer classification. Across subsets, the same four substantive profiles emerged, with comparable class proportions and high classification quality.

**Figure S12. Split-sample stability of the retained four-class latent profile solution across the training and testing subsets**

**Note.** Solid lines represent the testing subset and dashed lines represent the training subset. Points represent class-specific standardized means on the five HRI-15 dimensions. Profile labels were aligned across subsets on the basis of substantive profile content to facilitate comparison. Indicators were standardized in the full sample (*M* = 0, *SD* = 1). Within-class standard deviations were constrained to be equal across classes within each subset. In the training subset, the corresponding *SD*s were 0.585, 0.551, 0.810, 0.696, and 0.505 for Anthropophobia, Agoraphobia, Paranoia, Lethargy, and Depressive Mood, respectively; in the testing subset, they were 0.581, 0.538, 0.796, 0.712, and 0.491. The four profiles are: (1) Non-at-risk, (2) Depressive–lethargic, (3) At-risk, and (4) Social-anxiety/phobic.

**Supplementary CAT performance**

Detailed results of the ctree tuning analyses across the examined hyperparameter grid are reported below. Table S13 summarizes 5-fold cross-validation performance in the training set, whereas Table S14 reports the corresponding out-of-sample sensitivity results in the independent test set after refitting each configuration on the full training data. In both tables, ICC(A,1) and MAE are reported as aggregate indices across the six modeled outcomes (the HRI-15 total score and the five standardized subscale scores). Tables S15 and S16 report the corresponding outcome-specific results for the HRI-15 total score.

**Table** **S13. Five-fold cross-validation summary for all ctree hyperparameter configurations in the training set (aggregate indices across outcomes)**

| MinC | MinS | MinB | ICC(A,1) | MAE | % item saving | terminal leaves |
| --- | --- | --- | --- | --- | --- | --- |
| **0.10** | **10** | **10** | **0.818** | **1.014** | **54.876** | **278** |
| 0.20 | 10 | 10 | 0.818 | 1.014 | 54.876 | 278 |
| 0.50 | 10 | 10 | 0.818 | 1.014 | 54.876 | 278 |
| 0.70 | 10 | 10 | 0.818 | 1.014 | 54.876 | 278 |
| 0.90 | 10 | 10 | 0.818 | 1.014 | 54.906 | 276.6 |
| 0.10 | 20 | 10 | 0.818 | 1.014 | 54.876 | 278 |
| 0.20 | 20 | 10 | 0.818 | 1.014 | 54.876 | 278 |
| 0.50 | 20 | 10 | 0.818 | 1.014 | 54.876 | 278 |
| 0.70 | 20 | 10 | 0.818 | 1.014 | 54.876 | 278 |
| 0.90 | 20 | 10 | 0.818 | 1.014 | 54.906 | 276.6 |
| 0.10 | 20 | 20 | 0.798 | 1.085 | 60.816 | 138 |
| 0.20 | 20 | 20 | 0.798 | 1.085 | 60.816 | 138 |
| 0.50 | 20 | 20 | 0.798 | 1.085 | 60.816 | 138 |
| 0.70 | 20 | 20 | 0.798 | 1.085 | 60.816 | 138 |
| 0.90 | 20 | 20 | 0.798 | 1.085 | 60.816 | 138 |
| 0.10 | 50 | 10 | 0.794 | 1.092 | 58.57 | 150.4 |
| 0.20 | 50 | 10 | 0.794 | 1.092 | 58.57 | 150.4 |
| 0.50 | 50 | 10 | 0.794 | 1.092 | 58.57 | 150.4 |
| 0.70 | 50 | 10 | 0.794 | 1.092 | 58.57 | 150.4 |
| 0.90 | 50 | 10 | 0.794 | 1.092 | 58.57 | 150.4 |
| 0.10 | 50 | 20 | 0.794 | 1.099 | 61.225 | 128.8 |
| 0.20 | 50 | 20 | 0.794 | 1.099 | 61.225 | 128.8 |
| 0.50 | 50 | 20 | 0.794 | 1.099 | 61.225 | 128.8 |
| 0.70 | 50 | 20 | 0.794 | 1.099 | 61.225 | 128.8 |
| 0.90 | 50 | 20 | 0.794 | 1.099 | 61.225 | 128.8 |
| 0.10 | 50 | 50 | 0.766 | 1.193 | 66.704 | 58.2 |
| 0.20 | 50 | 50 | 0.766 | 1.193 | 66.704 | 58.2 |
| 0.50 | 50 | 50 | 0.766 | 1.193 | 66.704 | 58.2 |
| 0.70 | 50 | 50 | 0.766 | 1.193 | 66.704 | 58.2 |
| 0.90 | 50 | 50 | 0.766 | 1.193 | 66.704 | 58.2 |

**Note.** The table reports the mean results across the 5-fold cross validation for each candidate hyperparameter configuration. MinC = mincriterion, MinS = minsplit, MinB = minbucket, ICC(A,1) = two-way model, absolute agreement, single measure; MAE = mean absolute error. ICC(A,1) and MAE do not refer to the HRI-15 total score alone, but to the mean values across the six modeled outcomes. Percentage item saving refers to unique items needed to reach a leaf, rather than from terminal-node depth. The selected configuration is highlighted in bold.

**Table S14. Independent test-set sensitivity analysis for all ctree hyperparameter configurations (aggregate indices across outcomes)**

| MinC | MinS | MinB | ICC(A,1) | MAE | % item saving | Terminal leaves |
| --- | --- | --- | --- | --- | --- | --- |
| **0.10** | **10** | **10** | **0.834** | **0.967** | **53.139** | **349** |
| 0.20 | 10 | 10 | 0.834 | 0.967 | 53.139 | 349 |
| 0.50 | 10 | 10 | 0.834 | 0.967 | 53.139 | 349 |
| 0.70 | 10 | 10 | 0.834 | 0.967 | 53.139 | 349 |
| 0.90 | 10 | 10 | 0.834 | 0.967 | 53.153 | 347 |
| 0.10 | 20 | 10 | 0.834 | 0.967 | 53.139 | 349 |
| 0.20 | 20 | 10 | 0.834 | 0.967 | 53.139 | 349 |
| 0.50 | 20 | 10 | 0.834 | 0.967 | 53.139 | 349 |
| 0.70 | 20 | 10 | 0.834 | 0.967 | 53.139 | 349 |
| 0.90 | 20 | 10 | 0.834 | 0.967 | 53.153 | 347 |
| 0.10 | 20 | 20 | 0.813 | 1.043 | 59.631 | 176 |
| 0.20 | 20 | 20 | 0.813 | 1.043 | 59.631 | 176 |
| 0.50 | 20 | 20 | 0.813 | 1.043 | 59.631 | 176 |
| 0.70 | 20 | 20 | 0.813 | 1.043 | 59.631 | 176 |
| 0.90 | 20 | 20 | 0.813 | 1.043 | 59.631 | 176 |
| 0.10 | 50 | 10 | 0.811 | 1.038 | 56.952 | 187 |
| 0.20 | 50 | 10 | 0.811 | 1.038 | 56.952 | 187 |
| 0.50 | 50 | 10 | 0.811 | 1.038 | 56.952 | 187 |
| 0.70 | 50 | 10 | 0.811 | 1.038 | 56.952 | 187 |
| 0.90 | 50 | 10 | 0.811 | 1.038 | 56.952 | 187 |
| 0.10 | 50 | 20 | 0.808 | 1.06 | 60.215 | 156 |
| 0.20 | 50 | 20 | 0.808 | 1.06 | 60.215 | 156 |
| 0.50 | 50 | 20 | 0.808 | 1.06 | 60.215 | 156 |
| 0.70 | 50 | 20 | 0.808 | 1.06 | 60.215 | 156 |
| 0.90 | 50 | 20 | 0.808 | 1.06 | 60.215 | 156 |
| 0.10 | 50 | 50 | 0.782 | 1.163 | 65.764 | 65 |
| 0.20 | 50 | 50 | 0.782 | 1.163 | 65.764 | 65 |
| 0.50 | 50 | 50 | 0.782 | 1.163 | 65.764 | 65 |
| 0.70 | 50 | 50 | 0.782 | 1.163 | 65.764 | 65 |
| 0.90 | 50 | 50 | 0.782 | 1.163 | 65.764 | 65 |

**Note.** Each configuration was refitted on the full training set and then applied to the independent test set. The table reports the mean results. MinC = mincriterion, MinS = minsplit, MinB = minbucket, ICC(A,1) = two-way model, absolute agreement, MAE = mean absolute error. ICC(A,1) and MAE do not refer to the HRI-15 total score alone, but to the mean values across the six modeled outcomes. The selected configuration is highlighted in bold.

**Table S15. Five-fold cross-validation results for the HRI-15 total score across all ctree hyperparameter configurations in the training set**

| MinC | MinS | MinB | Mean *r* | SD *r* | Mean ICC(A,1) | *SD* ICC(A,1) | Mean MAE | *SD* MAE | Mean RMSE | *SD* RMSE |
| --- | --- | --- | --- | --- | --- | --- | --- | --- | --- | --- |
| **0.10** | **10** | **10** | **0.912** | **0.006** | **0.91** | **0.006** | **4.193** | **0.124** | **5.751** | **0.190** |
| 0.20 | 10 | 10 | 0.912 | 0.006 | 0.91 | 0.006 | 4.193 | 0.124 | 5.751 | 0.190 |
| 0.50 | 10 | 10 | 0.912 | 0.006 | 0.91 | 0.006 | 4.193 | 0.124 | 5.751 | 0.190 |
| 0.70 | 10 | 10 | 0.912 | 0.006 | 0.91 | 0.006 | 4.193 | 0.124 | 5.751 | 0.190 |
| 0.90 | 10 | 10 | 0.912 | 0.006 | 0.91 | 0.006 | 4.193 | 0.125 | 5.751 | 0.189 |
| 0.10 | 20 | 10 | 0.912 | 0.006 | 0.91 | 0.006 | 4.193 | 0.124 | 5.751 | 0.190 |
| 0.20 | 20 | 10 | 0.912 | 0.006 | 0.91 | 0.006 | 4.193 | 0.124 | 5.751 | 0.190 |
| 0.50 | 20 | 10 | 0.912 | 0.006 | 0.91 | 0.006 | 4.193 | 0.124 | 5.751 | 0.190 |
| 0.70 | 20 | 10 | 0.912 | 0.006 | 0.91 | 0.006 | 4.193 | 0.124 | 5.751 | 0.190 |
| 0.90 | 20 | 10 | 0.912 | 0.006 | 0.91 | 0.006 | 4.193 | 0.125 | 5.751 | 0.189 |
| 0.10 | 20 | 20 | 0.902 | 0.007 | 0.898 | 0.008 | 4.496 | 0.135 | 6.062 | 0.235 |
| 0.20 | 20 | 20 | 0.902 | 0.007 | 0.898 | 0.008 | 4.496 | 0.135 | 6.062 | 0.235 |
| 0.50 | 20 | 20 | 0.902 | 0.007 | 0.898 | 0.008 | 4.496 | 0.135 | 6.062 | 0.235 |
| 0.70 | 20 | 20 | 0.902 | 0.007 | 0.898 | 0.008 | 4.496 | 0.135 | 6.062 | 0.235 |
| 0.90 | 20 | 20 | 0.902 | 0.007 | 0.898 | 0.008 | 4.496 | 0.135 | 6.062 | 0.235 |
| 0.10 | 50 | 10 | 0.898 | 0.008 | 0.894 | 0.008 | 4.542 | 0.150 | 6.178 | 0.249 |
| 0.20 | 50 | 10 | 0.898 | 0.008 | 0.894 | 0.008 | 4.542 | 0.150 | 6.178 | 0.249 |
| 0.50 | 50 | 10 | 0.898 | 0.008 | 0.894 | 0.008 | 4.542 | 0.150 | 6.178 | 0.249 |
| 0.70 | 50 | 10 | 0.898 | 0.008 | 0.894 | 0.008 | 4.542 | 0.150 | 6.178 | 0.249 |
| 0.90 | 50 | 10 | 0.898 | 0.008 | 0.894 | 0.008 | 4.542 | 0.150 | 6.178 | 0.249 |
| 0.10 | 50 | 20 | 0.899 | 0.007 | 0.895 | 0.007 | 4.558 | 0.133 | 6.127 | 0.228 |
| 0.20 | 50 | 20 | 0.899 | 0.007 | 0.895 | 0.007 | 4.558 | 0.133 | 6.127 | 0.228 |
| 0.50 | 50 | 20 | 0.899 | 0.007 | 0.895 | 0.007 | 4.558 | 0.133 | 6.127 | 0.228 |
| 0.70 | 50 | 20 | 0.899 | 0.007 | 0.895 | 0.007 | 4.558 | 0.133 | 6.127 | 0.228 |
| 0.90 | 50 | 20 | 0.899 | 0.007 | 0.895 | 0.007 | 4.558 | 0.133 | 6.127 | 0.228 |
| 0.10 | 50 | 50 | 0.883 | 0.01 | 0.877 | 0.011 | 4.944 | 0.148 | 6.572 | 0.273 |
| 0.20 | 50 | 50 | 0.883 | 0.01 | 0.877 | 0.011 | 4.944 | 0.148 | 6.572 | 0.273 |
| 0.50 | 50 | 50 | 0.883 | 0.01 | 0.877 | 0.011 | 4.944 | 0.148 | 6.572 | 0.273 |
| 0.70 | 50 | 50 | 0.883 | 0.01 | 0.877 | 0.011 | 4.944 | 0.148 | 6.572 | 0.273 |
| 0.90 | 50 | 50 | 0.883 | 0.01 | 0.877 | 0.011 | 4.944 | 0.148 | 6.572 | 0.273 |

**Note.** The table reports outcome-specific results for the HRI-15 total score across 5-fold cross validation for each candidate hyperparameter configuration. For each performance index, both the mean and standard deviation across folds are shown. MinC = mincriterion, MinS = minsplit, MinB = minbucket, ICC(A,1) = two-way model, absolute agreement, MAE = mean absolute error, RMSE = root mean square error. The selected configuration is highlighted in bold.

**Table S16. Independent test-set results for the HRI-15 total score across all ctree hyperparameter configurations**

| MinC | MinS | MinB | *r* | ICC(A,1) | 95% CI for ICC | MAE | sMAE | RMSE | % item saving | Terminal leaves |
| --- | --- | --- | --- | --- | --- | --- | --- | --- | --- | --- |
| **0.10** | **10** | **10** | **0.925** | **0.922** | **[0.917, 0.928]** | **3.98** | **0.276** | **5.495** | **53.139** | **349** |
| 0.20 | 10 | 10 | 0.925 | 0.922 | [0.917, 0.928] | 3.98 | 0.276 | 5.495 | 53.139 | 349 |
| 0.50 | 10 | 10 | 0.925 | 0.922 | [0.917, 0.928] | 3.98 | 0.276 | 5.495 | 53.139 | 349 |
| 0.70 | 10 | 10 | 0.925 | 0.922 | [0.917, 0.928] | 3.98 | 0.276 | 5.495 | 53.139 | 349 |
| 0.90 | 10 | 10 | 0.925 | 0.922 | [0.917, 0.928] | 3.979 | 0.276 | 5.495 | 53.153 | 347 |
| 0.10 | 20 | 10 | 0.925 | 0.922 | [0.917, 0.928] | 3.98 | 0.276 | 5.495 | 53.139 | 349 |
| 0.20 | 20 | 10 | 0.925 | 0.922 | [0.917, 0.928] | 3.98 | 0.276 | 5.495 | 53.139 | 349 |
| 0.50 | 20 | 10 | 0.925 | 0.922 | [0.917, 0.928] | 3.98 | 0.276 | 5.495 | 53.139 | 349 |
| 0.70 | 20 | 10 | 0.925 | 0.922 | [0.917, 0.928] | 3.98 | 0.276 | 5.495 | 53.139 | 349 |
| 0.90 | 20 | 10 | 0.925 | 0.922 | [0.917, 0.928] | 3.979 | 0.276 | 5.495 | 53.153 | 347 |
| 0.10 | 20 | 20 | 0.913 | 0.909 | [0.902, 0.915] | 4.302 | 0.298 | 5.903 | 59.631 | 176 |
| 0.20 | 20 | 20 | 0.913 | 0.909 | [0.902, 0.915] | 4.302 | 0.298 | 5.903 | 59.631 | 176 |
| 0.50 | 20 | 20 | 0.913 | 0.909 | [0.902, 0.915] | 4.302 | 0.298 | 5.903 | 59.631 | 176 |
| 0.70 | 20 | 20 | 0.913 | 0.909 | [0.902, 0.915] | 4.302 | 0.298 | 5.903 | 59.631 | 176 |
| 0.90 | 20 | 20 | 0.913 | 0.909 | [0.902, 0.915] | 4.302 | 0.298 | 5.903 | 59.631 | 176 |
| 0.10 | 50 | 10 | 0.912 | 0.908 | [0.902, 0.914] | 4.285 | 0.297 | 5.909 | 56.952 | 187 |
| 0.20 | 50 | 10 | 0.912 | 0.908 | [0.902, 0.914] | 4.285 | 0.297 | 5.909 | 56.952 | 187 |
| 0.50 | 50 | 10 | 0.912 | 0.908 | [0.902, 0.914] | 4.285 | 0.297 | 5.909 | 56.952 | 187 |
| 0.70 | 50 | 10 | 0.912 | 0.908 | [0.902, 0.914] | 4.285 | 0.297 | 5.909 | 56.952 | 187 |
| 0.90 | 50 | 10 | 0.912 | 0.908 | [0.902, 0.914] | 4.285 | 0.297 | 5.909 | 56.952 | 187 |
| 0.10 | 50 | 20 | 0.91 | 0.906 | [0.899, 0.912] | 4.377 | 0.303 | 5.984 | 60.215 | 156 |
| 0.20 | 50 | 20 | 0.91 | 0.906 | [0.899, 0.912] | 4.377 | 0.303 | 5.984 | 60.215 | 156 |
| 0.50 | 50 | 20 | 0.91 | 0.906 | [0.899, 0.912] | 4.377 | 0.303 | 5.984 | 60.215 | 156 |
| 0.70 | 50 | 20 | 0.91 | 0.906 | [0.899, 0.912] | 4.377 | 0.303 | 5.984 | 60.215 | 156 |
| 0.90 | 50 | 20 | 0.91 | 0.906 | [0.899, 0.912] | 4.377 | 0.303 | 5.984 | 60.215 | 156 |
| 0.10 | 50 | 50 | 0.895 | 0.889 | [0.881, 0.897] | 4.801 | 0.333 | 6.438 | 65.764 | 65 |
| 0.20 | 50 | 50 | 0.895 | 0.889 | [0.881, 0.897] | 4.801 | 0.333 | 6.438 | 65.764 | 65 |
| 0.50 | 50 | 50 | 0.895 | 0.889 | [0.881, 0.897] | 4.801 | 0.333 | 6.438 | 65.764 | 65 |
| 0.70 | 50 | 50 | 0.895 | 0.889 | [0.881, 0.897] | 4.801 | 0.333 | 6.438 | 65.764 | 65 |
| 0.90 | 50 | 50 | 0.895 | 0.889 | [0.881, 0.897] | 4.801 | 0.333 | 6.438 | 65.764 | 65 |

**Note.** Each configuration was refitted on the full training set and then applied to the independent test set. The table reports outcome-specific results for the HRI-15 total score. For ICC(A,1), the corresponding 95% confidence interval is reported. MinC = mincriterion, MinS = minsplit, MinB = minbucket, ICC(A,1) = two-way model, absolute agreement (the corresponding 95% confidence interval is reported), MAE = mean absolute error, RMSE = root mean square error. Percentage item saving refers to unique items needed to reach a leaf, rather than from terminal-node depth. The selected configuration is highlighted in bold.

**Supplementary CAT classification and administration diagnostics**

Additional diagnostics for the retained adaptive solution are reported below. Table S17 summarizes cutoff-based classification performance for CAT-derived scores in the training and testing datasets, Figure S18 displays the corresponding calibration plot in the testing dataset, and Table S19 reports the associated calibration metrics. CAT administration characteristics are summarized in Table S20 and Figure S21, which report the distribution of administered items, and in Table S22, which reports item-by-item visitation frequencies. Table S23 provides a measurement-error-based summary of discrepancies between CAT-derived and full-length HRI-15 scores in the testing dataset. This section complements the full-length ROC results reported in Tables S1–S5 and Figure S6 and the CAT tuning results reported in Tables S13–S16. A summary of the leaf-level predictions is available in the Excel file “Supplementary table” on OSF (https://osf.io/3857s/overview?view_only=8f89e81a29544c8fb5f9a3a92c531e06).

**Table S17. Cutoff-based classification performance of CAT-derived HRI-15 scores in the training and testing datasets**

| Dataset | *N* | Positive cases | TP | FN | TN | FP | Sensitivity | Specificity | Accuracy | PPV | NPV |
| --- | --- | --- | --- | --- | --- | --- | --- | --- | --- | --- | --- |
| Training | 5866 | 374 | 273 | 101 | 4130 | 1362 | 0.730 | 0.752 | 0.751 | 0.167 | 0.976 |
| Testing | 2889 | 184 | 135 | 49 | 1971 | 734 | 0.734 | 0.729 | 0.729 | 0.155 | 0.976 |

**Note.** The table reports the classification performance obtained when the ROC-derived cutoff score (≥ 42) was applied to CAT-derived HRI-15 total scores. Results are shown separately for the training and testing datasets. TP = true positives; FN = false negatives; TN = true negatives; FP = false positives; PPV = positive predictive value; NPV = negative predictive value. The external criterion was the dichotomized self-confinement item used in the ROC analyses. Full-length cutoff performance is already reported in the Supplementary ROC section; accordingly, this table focuses specifically on CAT-derived classification.

**Figure S18. Calibration plot for CAT-derived HRI-15 scores in the testing dataset**

**Note.** Predicted probabilities were obtained by applying the logistic regression model estimated in the training dataset to the CAT-derived HRI-15 total scores in the testing dataset. Points represent grouped observed and predicted probabilities based on deciles of predicted risk. The diagonal line indicates ideal calibration. Closer correspondence between the calibration curve and the diagonal indicates better agreement between predicted risk and observed outcomes.

**Table S19. Calibration metrics for CAT-derived HRI-15 scores in the testing dataset**

| Score | *N* | Positive cases | Brier score | Calibration intercept | Calibration slope |
| --- | --- | --- | --- | --- | --- |
| CAT-derived  HRI-15 total score | 2889 | 184 | 0.0538 | -0.071 | 0.915 |

**Note.** The table reports calibration metrics for predicted criterion positivity derived from CAT-based HRI-15 total scores in the testing dataset. Predicted probabilities were obtained by applying the logistic regression model estimated in the training dataset to the testing dataset. The Brier score summarizes the overall discrepancy between predicted probabilities and observed outcomes, with lower values indicating better probabilistic accuracy. Ideal values are 0 for the calibration intercept and 1 for the calibration slope. Negative intercept values indicate a tendency to overestimate risk, whereas slopes below 1 indicate somewhat overly extreme predicted probabilities.

**Table S20. Distribution of administered items in the CAT across the training and testing datasets**

| Sample | *N* | Mean | *SD* | Min. | *P*25 | Median | *P*75 | Max. |
| --- | --- | --- | --- | --- | --- | --- | --- | --- |
| Training | 5866 | 7.03 | 2.99 | 3 | 5 | 7 | 9 | 14 |
| Testing | 2889 | 7.03 | 3.00 | 3 | 5 | 7 | 9 | 14 |

**Note.** The table summarizes the number of items administered by the CAT, including the sample size, mean, standard deviation, minimum, first quartile, median, third quartile, and maximum. Item savings are achieved because respondents receive only the items required along the adaptive decision path. The number of administered items reflects the true number of unique items used to reach the terminal leaf for each case.

**Figure S21. Distribution of the number of administered items in the CAT**

|  | Training | Testing |
| --- | --- | --- |
| 3 | 358 | 169 |
| 4 | 944 | 473 |
| 5 | 1105 | 564 |
| 6 | 481 | 228 |
| 7 | 825 | 403 |
| 8 | 567 | 262 |
| 9 | 396 | 208 |
| 10 | 367 | 153 |
| 11 | 188 | 114 |
| 12 | 170 | 90 |
| 13 | 160 | 83 |
| 14 | 305 | 142 |

**Note.** The figure displays the frequency distribution of the number of items administered by the CAT. Results are shown separately for the training and testing datasets. The distribution illustrates how the reduction in administration burden was achieved across respondents rather than only on average. Lower values indicate shorter adaptive administrations.

**Table S22. Item-by-item visitation frequencies in the CAT for the testing dataset**

| Item | Cases visited | Proportion visited |
| --- | --- | --- |
| Item13 | 2889 | 1.000 |
| Item14 | 2889 | 1.000 |
| Item15 | 2889 | 1.000 |
| Item4 | 1962 | 0.679 |
| Item7 | 1491 | 0.516 |
| Item2 | 1443 | 0.499 |
| Item11 | 1397 | 0.484 |
| Item8 | 1067 | 0.369 |
| Item5 | 892 | 0.309 |
| Item6 | 632 | 0.219 |
| Item3 | 630 | 0.218 |
| Item12 | 607 | 0.210 |
| Item9 | 566 | 0.196 |
| Item1 | 501 | 0.173 |
| Item10 | 452 | 0.156 |

**Note.** The table reports how often each HRI-15 item was visited during adaptive administration in the testing dataset. Frequencies refer to the number and proportion of cases whose adaptive path required the corresponding item. Higher values indicate items that were more frequently used by the decision tree to route respondents toward terminal leaves. These frequencies reflect the operational importance of items within the fitted CAT structure, not item difficulty or endorsement rates.

**Table S23. Measurement-error-based discrepancy summary for CAT-derived versus full-length HRI-15 scores in the testing dataset**

| Outcome | Mean full length score | Mean CAT score | Mean absolute difference | Cronbach’s *α* | *SD* train | SEM | SEdiff | Threshold 95% | *N* exceeding 95 % threshold | Proportion exceeding 95 % thresholds |
| --- | --- | --- | --- | --- | --- | --- | --- | --- | --- | --- |
| Sum score HRI-15 | 34.72 | 34.654 | 3.98 | 0.916 | 14.021 | 4.06 | 5.741 | 11.253 | 167 | 0.058 |
| Anthropophobia | 0.021 | 0.023 | 0.445 | 0.837 | 0.995 | 0.402 | 0.568 | 1.114 | 273 | 0.094 |
| Agoraphobia | 0.018 | 0.004 | 0.42 | 0.827 | 0.993 | 0.413 | 0.584 | 1.145 | 212 | 0.073 |
| Paranoia | 0.009 | 0.013 | 0.466 | 0.763 | 0.999 | 0.486 | 0.687 | 1.347 | 132 | 0.046 |
| Lethargy | 0.029 | 0.017 | 0.465 | 0.821 | 0.991 | 0.419 | 0.592 | 1.161 | 247 | 0.085 |
| Depressive Mood | 0.024 | 0.023 | 0.025 | 0.779 | 0.994 | 0.467 | 0.66 | 1.294 | 000 | 0.000 |

**Note.** The table summarizes disagreement between CAT-derived and full-length scores using a measurement-error-based approach. For each outcome, the standard error of measurement (SEM) was computed as the scale *SD* × $\sqrt{(1 - \alpha)}$, where *SD* refers to the standard deviation of the full-length score and *α* refers to the scale reliability based on Cronbach’s alpha coefficients computed on the training dataset. The standard error of the difference (SEdiff) was computed as $\sqrt{2}$ × SEM, and the 95% discrepancy threshold was defined as 1.96 × SEdiff. Cases whose absolute CAT–full-length difference exceeded this threshold were flagged as showing a meaningful discrepancy on the basis of measurement error. Proportions are reported relative to the size of cases in the testing dataset (*N* = 2889).

**Concluding note**

Overall, these supplementary analyses provide a more comprehensive picture of the classification validity, administration characteristics, and methodological robustness of the proposed adaptive procedure. They show that the CAT maintains satisfactory classification performance and calibration, achieves substantial administration efficiency, and remains robust across the examined tuning configurations. They also indicate that discrepancies between CAT-derived and full-length scores were generally limited across outcomes. Taken together, these findings strengthen confidence in the adequacy of the procedure while also highlighting the importance of future research aimed at understanding more directly the circumstances under which uncommon response patterns may lead to larger CAT–full-length deviations, especially in real-world and longitudinal applications.
