# Supplementary material for: Interpretable machine learning for hikikomori screening: The adaptive HRI-15
Source: PLoS One. 2026 Aug 20;21(8):e0355595. doi: 10.1371/journal.pone.0355595 (PMC13492808; doi:10.1371/journal.pone.0355595)
Supplement: Supplementary file 2 [file pone.0355595.s002.docx]

**Responses to Reviewers**

*We sincerely thank the Editor and the Reviewers for their careful evaluation of our manuscript and for their thoughtful and constructive comments. We considered all suggestions with great attention and revised the manuscript accordingly. We believe that these revisions have significantly strengthened the paper. All changes in the revised version are highlighted in light blue. Below, we provide a detailed point-by-point response to each comment.*

Reviewer #1

The manuscript addresses an important applied problem, uses an exceptionally large, nationally sampled data set, and combines appropriate analytic techniques (ROC analysis, latent profile analysis, and interpretable ML–based CAT). The methodological approach is coherent and the translational component (a Shiny application; code link on OSF) is commendable. However, several substantive issues must be addressed prior to publication to ensure conceptual accuracy, analytic robustness, and compliance with journal policies.

*We sincerely thank the Reviewer for these positive comments and constructive suggestions. We greatly appreciate the recognition of the strengths of the study and have carefully considered all points raised in revising the manuscript.*

1.The manuscript states the data are owned by the Italian National Institute of Health (Istituto Superiore di Sanità) and are not publicly available. This does not satisfy PLOS ONE’s data availability requirements. The authors must either:
a) deposit a suitably de-identified version of the dataset in a public or controlled-access repository with documented access procedures, or
b) provide a clear, formal controlled-access mechanism (repository name, contact, application procedure, and expected time to obtain access) that permits independent verification while protecting participant privacy, and include a justification that satisfies PLOS exception criteria. If legal/ethical constraints prevent sharing item-level data, the authors should provide synthetic data or aggregated tables sufficient to reproduce key results and supply all analysis scripts (complete R / Mplus code) together with the Shiny app configuration files.

*We thank the Reviewer for this suggestion. A de-identified version of the dataset has been made publicly available on: https://osf.io/3857s/overview?view_only=8f89e81a29544c8fb5f9a3a92c531e06*

2.The ROC cutoff is derived using a single self-report item about room confinement. That item is conceptually close to HRI content and therefore may bias classification (criterion contamination). The authors should:

Provide justification for using that single item as the external criterion and discuss its limitations explicitly; and, where possible, validate the cutoff against an independent clinical benchmark (e.g., structured clinical interview or clinician rating) or at least against external correlates (functional impairment, school absence).
Report ROC confidence intervals, decision thresholds sensitivity analyses (different dichotomizations), and calibration metrics.

*We sincerely thank the Reviewer for this constructive comment. In the revised version of the manuscript, the rationale for using the room-confinement item as the external criterion has been better clarified and its limitations have been discussed more explicitly. In particular, the revised version of Introduction states more clearly that this item was selected because it captures a core behavioral manifestation of prolonged social withdrawal and extends previous HRI-15 work by relying on a more detailed, frequency-based criterion rather than on a simple binary indicator.*

*The ROC-related reporting has also been substantially expanded. Specifically, the revised manuscript reports confidence intervals for ROC performance indices, a sensitivity analysis based on a reasonable and more restrictive alternative dichotomization of the criterion (primary coding: responses 4–5 [“Every week, but not every day” / “Every day”] as positive; stricter coding: only 5 [“Every day”] as positive) and calibration diagnostics derived from logistic regression models estimated in the training set and applied to the testing set. These additions include confusion matrices, Brier scores, calibration intercepts and slopes, and calibration plots, reported in the main text and in the Supplementary Materials (main text: ROC Results section; Supplementary Tables S1–S5 and Figure S6).*

*In the revised Discussion, it is stated more explicitly that the external criterion remains a single self-report indicator rather than an independent clinical diagnosis, and that this may have introduced some degree of criterion misclassification, with possible implications for sensitivity, specificity, and the location of the optimal cutoff. The resulting threshold is therefore framed as a pragmatic screening indicator rather than as a definitive diagnostic benchmark.*

*Validation against an independent clinical benchmark (e.g., a structured interview or clinician rating) was not possible because such data were not available in the present survey. This limitation has been explicitly acknowledged, and future studies are indicated as needing to extend validation through clinical assessments, multi-informant information, and additional external indicators of functional impairment. It should nevertheless be noted that, although an independent clinical benchmark was unavailable, the observed associations between LPA-derived profiles, the external criterion, and ROC-based classification provided convergent support for the clinical meaningfulness of the identified threshold (Table 4).*

3.The train/test split is appropriate but insufficient alone. Please provide one or more of the following robustness checks: repeated k-fold cross-validation, bootstrap validation, or external temporal/geographic validation (if available).
Justify hyperparameter choices for the conditional-inference tree (mincriterion/minsplit/minbucket). Provide sensitivity analyses showing how results (e.g., item savings, ICC, MAE) change when tuning these parameters.
Compare the proposed CART/ctree-based CAT with at least one alternative (for example: an IRT-based CAT, regularized regression, or random-forest surrogate) to demonstrate that the chosen approach balances interpretability and accuracy.

*We really appreciate the comment from the Reviewer. In the revised version of the manuscript, the rationale for the selected ctree configuration has been clarified more explicitly, and the tuning results have been reported in greater detail. Specifically, candidate values for mincriterion, minsplit, and minbucket were examined over a broad grid of plausible settings, first through 5-fold cross-validation within the training subset and then through refitting on the full training set followed by evaluation on the independent test set. Moreover, it was further clarified that the selected configuration was retained because it provided the most appropriate balance between predictive accuracy and administration efficiency.*

*This allowed the sensitivity of agreement, prediction error, item savings, and tree complexity to alternative settings to be examined systematically. These additions are described in the main text (Method Section, Subsection “Machine-Learning Computerized Adaptive Test (CAT)”, Sub-subsection “CAT development”), and the detailed tuning and sensitivity results are reported in the Supplementary Materials (Tables S13–S16).*

*Although repeated k-fold cross-validation, bootstrap validation, or external temporal/geographic validation would certainly be valuable extensions, these analyses were not feasible within the scope of the present study. Nevertheless, robustness beyond a simple training/test split was addressed through internal cross-validation for model selection, independent test-set evaluation, and systematic sensitivity analyses across the examined hyperparameter grid.*

*A comparison with alternative approaches was also considered carefully. However, regularized regression and random-forest surrogates were judged to be less suitable as primary comparators because they do not naturally support intrinsically adaptive administration and are therefore not fully aligned with the main goal of the study, namely the development of an interpretable CAT for real-time adaptive administration. An IRT-based CAT would certainly represent an important benchmark and a highly relevant direction for future work; however, including such a comparison would have required introducing a substantially broader psychometric framework, with additional modeling assumptions and implementation choices, and would likely have moved the manuscript beyond its current focus. For this reason, the revision prioritizes a clearer justification of the selected ctree-based approach together with expanded robustness and sensitivity evidence.*

4.The manuscript selects a four-class solution but reports significant VLMR/LMR p-values up to 5 classes. Provide a clearer, reproducible rationale for selecting four classes: include class stability checks (e.g., split-sample LPA), entropy and posterior probabilities per class, and substantive interpretability criteria.
Report class-specific sizes and standard errors clearly, and include the full fit-statistics table in supplement. Consider reporting model-based probabilities and provide a diagram/table with profile means and variance.

*We sincerely thank the Reviewer for this important comment. In the revised version of the manuscript, the rationale for retaining the four-class latent profile solution has been clarified more explicitly and documented in greater detail. Specifically, the full fit-statistics table for the one- to five-class solutions is reported in the main text (Table 1), and the retained solution is justified on the basis of a joint evaluation of statistical fit, classification quality, class size, and substantive interpretability. Although the VLMR/LMR tests remained significant up to the five-class solution, the improvement from four to five classes was comparatively limited, whereas the four-class model showed the highest entropy and yielded a clearer and more interpretable configuration with well-defined class proportions. For this reason, the selected solution was framed as the best balance among fit improvement, classification quality, parsimony, and substantive interpretability. Additional classification diagnostics are reported in the Supplementary Materials, including estimated and modal class counts and proportions (Table S7), average posterior probabilities (Table S8), class-specific indicator means and standard errors (Table S9), within-class variance parameters (Table S10), and split-sample stability analyses across the training and testing subsets (Table S11 and Figure S12). These additions were intended to make the rationale for the retained four-class solution more transparent and reproducible.*

5. Correct the in-text statement implying hikikomori is a formal DSM-5 diagnosis. The condition is a socio-cultural syndrome under scholarly debate and is not listed as a discrete DSM-5 disorder. Reframe language accordingly and cite current nosological discussions.

*We sincerely thank the Reviewer for this comment. Based on this comment and on the relevant literature, the framing of hikikomori in the revised manuscript has been refined. In particular, the term is no longer presented as a formal diagnostic category in the DSM-5 or DSM-5-TR, but rather as a clinically relevant and culturally inflected syndrome/condition whose nosological status remains under scholarly debate. The revised text also specifies that hikikomori is mentioned in the DSM-5/DSM-5-TR in relation to cultural concepts of distress, but is not recognized as a discrete DSM-5/DSM-5-TR disorder. Current references addressing this nosological issue have also been added in the Introduction section.*

6. Include confusion matrices, calibration plots, and calibration statistics for cutoff-based classification (both full-length and CAT-derived). Provide ICC tables with 95% confidence intervals.

For ML/CAT performance: report distributions of administered items, item-by-item visitation frequencies, and leaf-level prediction summaries (means, SD, n). Provide examples of cases where CAT and full-length scores diverged and explain why.

*We thank the Reviewer for this comment. The requested classification and CAT-performance diagnostics have been substantially expanded in the revised version of the manuscript and in the Supplementary Materials. Specifically, confusion matrices, calibration statistics, and calibration plots are reported for both the full-length and CAT-derived classifications (full-length: Supplementary Tables S1–S5 and Figure S6; CAT-derived: Supplementary Tables S17–S19 and Figure S18). In addition, ICC values with 95% confidence intervals are reported for the agreement between CAT-derived and full-length scores (main text: Table 5; Supplementary Table S16 for the HRI-15 total score across tuning configurations).*

*With regard to CAT administration characteristics, the Supplementary Materials report the distribution of administered items (Supplementary Table S20 and Figure S21), item-by-item visitation frequencies (Supplementary Table S22), and leaf-level prediction summaries, which are provided in an Excel file on the OSF project, as indicated in the Supplementary Materials.*

*As for discrepancies between CAT-derived and full-length scores, the revised materials include a measurement-error-based summary of disagreement patterns across outcomes (Supplementary Table S23). Rather than adding a small set of illustrative single cases, an aggregate summary was preferred in order to avoid overinterpretation of potentially idiosyncratic observations. The reported results indicate that CAT–full-length discrepancies were generally limited across outcomes, as assessed through a measurement-error–based comparison using SEM-derived thresholds for meaningful score differences. A brief statement has therefore been added to the Supplementary Materials to clarify this point and to note that future research should examine more directly the conditions under which uncommon response patterns may yield larger CAT–full-length deviations.*

Make all analysis code and the exact Shiny app tree/dictionary files available on the OSF project; include a README with instructions to reproduce the analyses and to run the Shiny app locally.

*We thank the Reviewer for this suggestion. All analysis code, the Shiny app tree file, and related README files have been made available on OSF: https://osf.io/3857s/overview?view_only=8f89e81a29544c8fb5f9a3a92c531e06*

Reviewer #2:

The manuscript presents a well-structured and methodologically rigorous study on the development of an interpretable machine learning–based computerized adaptive test (CAT) for hikikomori screening using the HRI-15. The topic is timely and relevant, and the integration of psychometric modeling with machine learning represents a meaningful contribution to both clinical assessment and large-scale screening.

A key strength of the study is the use of a large, nationally representative sample (N = 8,755), which provides strong statistical power and enhances the generalizability of the findings. The analytical framework is robust and appropriately implemented, combining ROC analysis, latent profile analysis (LPA), and a conditional inference tree–based CAT model. The use of a training/test split and multiple evaluation metrics (e.g., AUC, entropy, ICC, Cohen’s κ) demonstrates good methodological rigor and supports the validity of the conclusions.

The identification of a clinically interpretable cut-off score (≥ 42) and the derivation of four meaningful latent profiles represent valuable contributions, particularly for applied settings where both screening and personalization are needed. The CAT implementation is also well justified and shows substantial efficiency gains, reducing item administration while maintaining strong agreement with full-length scores. The inclusion of a Shiny-based application further enhances the practical utility of the work.

*We sincerely thank the Reviewer for these thoughtful and constructive comments, and for the overall positive evaluation of the manuscript. The points raised were carefully considered in the revised version, and the corresponding changes are highlighted in light blue.*

Despite these strengths, there are a few points that should be addressed to further strengthen the manuscript:

External Criterion for ROC Analysis:

The ROC analysis relies on a single self-report item as the external criterion for hikikomori risk classification. While this approach is understandable in large-scale surveys, it may introduce measurement limitations. The authors should discuss the potential impact of this choice on classification accuracy and consider referencing validation against clinical diagnoses where possible.

*We thank the Reviewer for this important observation. In the revised version of the manuscript, the rationale for using the room-confinement item as the external criterion has been clarified more fully and its limitations have been discussed more explicitly. In particular, the revised Introduction explains more clearly that this item was selected because it captures a core behavioral manifestation of prolonged social withdrawal and extends previous HRI-15 work by relying on a more detailed, frequency-based criterion rather than on a simple binary indicator.*

*The revised Discussion also makes clearer that the external criterion remains a single self-report indicator rather than an independent clinical diagnosis, and that this may have introduced some degree of criterion misclassification, with possible implications for sensitivity, specificity, and the location of the optimal cutoff. Accordingly, the resulting threshold is framed as a pragmatic screening indicator rather than as a definitive diagnostic benchmark.*

*Validation against an independent clinical benchmark (e.g., a structured interview or clinician rating) was not possible because such data were not available in the present survey. This limitation has been acknowledged explicitly, and future studies are indicated as needing to extend validation through clinical assessments, multi-informant information, and additional external indicators of functional impairment. At the same time, the observed associations between LPA-derived profiles, the external criterion, and ROC-based classification were consistent with the clinical meaningfulness of the identified threshold (Table 4).*

*Additional revisions on this topic were also introduced in response to Reviewer 1, including further ROC-related reporting and supplementary diagnostic information. For additional details, please see the response to Reviewer 1, Comment 2.*

Longitudinal or Real-World Validation:

The study demonstrates strong cross-sectional performance. Future work could explore longitudinal validation or real-world deployment to assess how the CAT performs over time and in applied clinical or school settings.

*This point has also been incorporated more clearly into the Discussion. The revised manuscript explicitly acknowledges that the present study demonstrates strong cross-sectional performance, while also indicating that future research should evaluate the CAT in longitudinal and real-world applications. Specifically, the revised text highlights the need to assess whether CAT-derived classifications remain stable over time, whether the instrument is sensitive to change, and how it performs when embedded in routine school- or clinic-based screening workflows and under real-world administration conditions.*

Model Interpretability and Clinical Use:

The use of conditional inference trees is a strength in terms of interpretability. However, the manuscript would benefit from a clearer illustration or example of the decision path (e.g., a sample tree or decision rules) to help clinicians better understand how the adaptive process operates in practice.

*We thank the Reviewer for this useful suggestion. To improve the interpretability of the adaptive procedure for applied readers, the revised manuscript includes a schematic illustration of the ctree procedure and adaptive routing in the Methods section (Figure 1). This solution was preferred because the full tree, given its size and complexity, is not easily readable as an in-text figure. By contrast, the new figure is more informative for the reader, as it illustrates both the tree-building procedure and the logic of adaptive administration in two complementary panels, thereby providing a clearer overview of how the method was developed and how the adaptive decision process operates in practice*.

Data Availability and Reproducibility:

The manuscript states that the data are not publicly available due to privacy restrictions. While this is understandable, the authors are encouraged to provide as much transparency as possible, for example by sharing synthetic datasets, detailed preprocessing steps, or expanded documentation of the analysis pipeline to facilitate reproducibility.

*We thank the Reviewer for this suggestion. A de-identified version of the dataset and the analysis code have been made publicly available on: https://osf.io/3857s/overview?view_only=8f89e81a29544c8fb5f9a3a92c531e06*
