# Supplementary material for: Interpretable machine learning for hikikomori screening: The adaptive HRI-15
Source: PLoS One. 2026 Aug 20;21(8):e0355595. doi: 10.1371/journal.pone.0355595 (PMC13492808; doi:10.1371/journal.pone.0355595)
Supplement: Peer review history

# Peer review: Decision Letter 0

**DOI:** 10.1371/journal.pone.0355595.r001

**Authors:** Boshra Arnout

23 Mar 2026

Dear Dr. Anselmi,

Thank you for submitting your manuscript to PLOS ONE. After careful consideration, we feel that it has merit but does not fully meet PLOS ONE’s publication criteria as it currently stands. Therefore, we invite you to submit a revised version of the manuscript that addresses the points raised during the review process.

Please submit your revised manuscript by May 07 2026 11:59PM. If you will need more time than this to complete your revisions, please reply to this message or contact the journal office at plosone@plos.org. When you're ready to submit your revision, log on to https://www.editorialmanager.com/pone/ and select the 'Submissions Needing Revision' folder to locate your manuscript file.

- A letter that responds to each point raised by the academic editor and reviewer(s). You should upload this letter as a separate file labeled 'Response to Reviewers'.
- A marked-up copy of your manuscript that highlights changes made to the original version. You should upload this as a separate file labeled 'Revised Manuscript with Track Changes'.
- An unmarked version of your revised paper without tracked changes. You should upload this as a separate file labeled 'Manuscript'.

If you would like to make changes to your financial disclosure, please include your updated statement in your cover letter. Guidelines for resubmitting your figure files are available below the reviewer comments at the end of this letter.

If applicable, we recommend that you deposit your laboratory protocols in protocols.io to enhance the reproducibility of your results. Protocols.io assigns your protocol its own identifier (DOI) so that it can be cited independently in the future. For instructions see: https://journals.plos.org/plosone/s/submission-guidelines#loc-laboratory-protocols. Additionally, PLOS ONE offers an option for publishing peer-reviewed Lab Protocol articles, which describe protocols hosted on protocols.io. Read more information on sharing protocols at https://plos.org/protocols?utm_medium=editorial-email&utm_source=authorletters&utm_campaign=protocols.

We look forward to receiving your revised manuscript.

Kind regards,

Boshra A. Arnout, Professor

Academic Editor

PLOS One

Journal Requirements:

When submitting your revision, we need you to address these additional requirements.

1. Please ensure that your manuscript meets PLOS ONE's style requirements, including those for file naming. The PLOS ONE style templates can be found at

https://journals.plos.org/plosone/s/file?id=wjVg/PLOSOne_formatting_sample_main_body.pdf and

https://journals.plos.org/plosone/s/file?id=ba62/PLOSOne_formatting_sample_title_authors_affiliations.pdf

2. Please note that PLOS One has specific guidelines on code sharing for submissions in which author-generated code underpins the findings in the manuscript. In these cases, we expect all author-generated code to be made available without restrictions upon publication of the work.

Please review our guidelines at https://journals.plos.org/plosone/s/materials-and-software-sharing#loc-sharing-code and ensure that your code is shared in a way that follows best practice and facilitates reproducibility and reuse.

3. Thank you for stating the following financial disclosure:

“This study received financial support from Department for Antidrug Policies [collaboration agreement CA09, 2021]”

Please state what role the funders took in the study.  If the funders had no role, please state: "The funders had no role in study design, data collection and analysis, decision to publish, or preparation of the manuscript." If this statement is not correct you must amend it as needed.

Please include this amended Role of Funder statement in your cover letter; we will change the online submission form on your behalf.

4. Please note that funding information should not appear in the Acknowledgments section or other areas of your manuscript. We will only publish funding information present in the Funding Statement section of the online submission form. Please remove any funding-related text from the manuscript.

5. For studies involving third-party data, we encourage authors to share any data specific to their analyses that they can legally distribute. PLOS recognizes, however, that authors may be using third-party data they do not have the rights to share. When third-party data cannot be publicly shared, authors must provide all information necessary for interested researchers to apply to gain access to the data. (https://journals.plos.org/plosone/s/data-availability#loc-acceptable-data-access-restrictions)

For any third-party data that the authors cannot legally distribute, they should include the following information in their Data Availability Statement upon submission:

1) A description of the data set and the third-party source

2) If applicable, verification of permission to use the data set

3) Confirmation of whether the authors received any special privileges in accessing the data that other researchers would not have

4) All necessary contact information others would need to apply to gain access to the data

6. Thank you for stating the following in the Competing Interests section:

“The authors have declared that no competing interests exist.”

We note that one or more of the authors are employed by a commercial company: Explora Research and Statistical Analysis or Centro Explora

1) Please provide an amended Funding Statement declaring this commercial affiliation, as well as a statement regarding the Role of Funders in your study. If the funding organization did not play a role in the study design, data collection and analysis, decision to publish, or preparation of the manuscript and only provided financial support in the form of authors' salaries and/or research materials, please review your statements relating to the author contributions, and ensure you have specifically and accurately indicated the role(s) that these authors had in your study. You can update author roles in the Author Contributions section of the online submission form.

Please also include the following statement within your amended Funding Statement.

“The funder provided support in the form of salaries for authors [insert relevant initials], but did not have any additional role in the study design, data collection and analysis, decision to publish, or preparation of the manuscript. The specific roles of these authors are articulated in the ‘author contributions’ section.”

If your commercial affiliation did play a role in your study, please state and explain this role within your updated Funding Statement.

2) Please also provide an updated Competing Interests Statement declaring this commercial affiliation along with any other relevant declarations relating to employment, consultancy, patents, products in development, or marketed products, etc.

Within your Competing Interests Statement, please confirm that this commercial affiliation does not alter your adherence to all PLOS ONE policies on sharing data and materials by including the following statement: ""This does not alter our adherence to  PLOS ONE policies on sharing data and materials.” (as detailed online in our guide for authors http://journals.plos.org/plosone/s/competing-interests) . If this adherence statement is not accurate and  there are restrictions on sharing of data and/or materials, please state these. Please note that we cannot proceed with consideration of your article until this information has been declared.

Please include both an updated Funding Statement and Competing Interests Statement in your cover letter. We will change the online submission form on your behalf.

7. Please include your full ethics statement in the ‘Methods’ section of your manuscript file. In your statement, please include the full name of the IRB or ethics committee who approved or waived your study, as well as whether or not you obtained informed written or verbal consent. If consent was waived for your study, please include this information in your statement as well.

8. We note you have included a table to which you do not refer in the text of your manuscript. Please ensure that you refer to Table 4 in your text; if accepted, production will need this reference to link the reader to the Table.

9. If the reviewer comments include a recommendation to cite specific previously published works, please review and evaluate these publications to determine whether they are relevant and should be cited. There is no requirement to cite these works unless the editor has indicated otherwise.

[Note: HTML markup is below. Please do not edit.]

Reviewers' comments:

Reviewer's Responses to Questions

**Comments to the Author**

1. Is the manuscript technically sound, and do the data support the conclusions?

Reviewer #1: Yes

Reviewer #2: Yes

**********

2. Has the statistical analysis been performed appropriately and rigorously?

Reviewer #1: Yes

Reviewer #2: Yes

**********

3. Have the authors made all data underlying the findings in their manuscript fully available?

The PLOS Data policy

Reviewer #1: No

Reviewer #2: No

**********

4. Is the manuscript presented in an intelligible fashion and written in standard English?

Reviewer #1: Yes

Reviewer #2: Yes

**********

Reviewer #1: The manuscript addresses an important applied problem, uses an exceptionally large, nationally sampled data set, and combines appropriate analytic techniques (ROC analysis, latent profile analysis, and interpretable ML–based CAT). The methodological approach is coherent and the translational component (a Shiny application; code link on OSF) is commendable. However, several substantive issues must be addressed prior to publication to ensure conceptual accuracy, analytic robustness, and compliance with journal policies.

1.The manuscript states the data are owned by the Italian National Institute of Health (Istituto Superiore di Sanità) and are not publicly available. This does not satisfy PLOS ONE’s data availability requirements. The authors must either:

a) deposit a suitably de-identified version of the dataset in a public or controlled-access repository with documented access procedures, or

b) provide a clear, formal controlled-access mechanism (repository name, contact, application procedure, and expected time to obtain access) that permits independent verification while protecting participant privacy, and include a justification that satisfies PLOS exception criteria.

If legal/ethical constraints prevent sharing item-level data, the authors should provide synthetic data or aggregated tables sufficient to reproduce key results and supply all analysis scripts (complete R / Mplus code) together with the Shiny app configuration files.

2.The ROC cutoff is derived using a single self-report item about room confinement. That item is conceptually close to HRI content and therefore may bias classification (criterion contamination). The authors should:

Provide justification for using that single item as the external criterion and discuss its limitations explicitly; and, where possible, validate the cutoff against an independent clinical benchmark (e.g., structured clinical interview or clinician rating) or at least against external correlates (functional impairment, school absence).

Report ROC confidence intervals, decision thresholds sensitivity analyses (different dichotomizations), and calibration metrics.

3.The train/test split is appropriate but insufficient alone. Please provide one or more of the following robustness checks: repeated k-fold cross-validation, bootstrap validation, or external temporal/geographic validation (if available).

Justify hyperparameter choices for the conditional-inference tree (mincriterion/minsplit/minbucket). Provide sensitivity analyses showing how results (e.g., item savings, ICC, MAE) change when tuning these parameters.

Compare the proposed CART/ctree-based CAT with at least one alternative (for example: an IRT-based CAT, regularized regression, or random-forest surrogate) to demonstrate that the chosen approach balances interpretability and accuracy.

4.The manuscript selects a four-class solution but reports significant VLMR/LMR p-values up to 5 classes. Provide a clearer, reproducible rationale for selecting four classes: include class stability checks (e.g., split-sample LPA), entropy and posterior probabilities per class, and substantive interpretability criteria.

Report class-specific sizes and standard errors clearly, and include the full fit-statistics table in supplement. Consider reporting model-based probabilities and provide a diagram/table with profile means and variance.

5.Correct the in-text statement implying hikikomori is a formal DSM-5 diagnosis. The condition is a socio-cultural syndrome under scholarly debate and is not listed as a discrete DSM-5 disorder. Reframe language accordingly and cite current nosological discussions.

6.Include confusion matrices, calibration plots, and calibration statistics for cutoff-based classification (both full-length and CAT-derived). Provide ICC tables with 95% confidence intervals.

For ML/CAT performance: report distributions of administered items, item-by-item visitation frequencies, and leaf-level prediction summaries (means, SD, n). Provide examples of cases where CAT and full-length scores diverged and explain why.

Make all analysis code and the exact Shiny app tree/dictionary files available on the OSF project; include a README with instructions to reproduce the analyses and to run the Shiny app locally.

Reviewer #2: The manuscript presents a well-structured and methodologically rigorous study on the development of an interpretable machine learning–based computerized adaptive test (CAT) for hikikomori screening using the HRI-15. The topic is timely and relevant, and the integration of psychometric modeling with machine learning represents a meaningful contribution to both clinical assessment and large-scale screening.

A key strength of the study is the use of a large, nationally representative sample (N = 8,755), which provides strong statistical power and enhances the generalizability of the findings. The analytical framework is robust and appropriately implemented, combining ROC analysis, latent profile analysis (LPA), and a conditional inference tree–based CAT model. The use of a training/test split and multiple evaluation metrics (e.g., AUC, entropy, ICC, Cohen’s κ) demonstrates good methodological rigor and supports the validity of the conclusions.

The identification of a clinically interpretable cut-off score (≥ 42) and the derivation of four meaningful latent profiles represent valuable contributions, particularly for applied settings where both screening and personalization are needed. The CAT implementation is also well justified and shows substantial efficiency gains, reducing item administration while maintaining strong agreement with full-length scores. The inclusion of a Shiny-based application further enhances the practical utility of the work.

Despite these strengths, there are a few points that should be addressed to further strengthen the manuscript:

External Criterion for ROC Analysis:

The ROC analysis relies on a single self-report item as the external criterion for hikikomori risk classification. While this approach is understandable in large-scale surveys, it may introduce measurement limitations. The authors should discuss the potential impact of this choice on classification accuracy and consider referencing validation against clinical diagnoses where possible.

Generalizability Across Cultures and Age Groups:

The sample consists of Italian adolescents. Given that hikikomori is a culturally influenced phenomenon, the authors should elaborate on the extent to which the findings and the derived CAT model can generalize to other cultural contexts and age groups.

Data Availability and Reproducibility:

The manuscript states that the data are not publicly available due to privacy restrictions. While this is understandable, the authors are encouraged to provide as much transparency as possible, for example by sharing synthetic datasets, detailed preprocessing steps, or expanded documentation of the analysis pipeline to facilitate reproducibility.

Model Interpretability and Clinical Use:

The use of conditional inference trees is a strength in terms of interpretability. However, the manuscript would benefit from a clearer illustration or example of the decision path (e.g., a sample tree or decision rules) to help clinicians better understand how the adaptive process operates in practice.

Longitudinal or Real-World Validation:

The study demonstrates strong cross-sectional performance. Future work could explore longitudinal validation or real-world deployment to assess how the CAT performs over time and in applied clinical or school settings.

In conclusion, this manuscript is technically sound, well written, and provides a valuable contribution to the field. Addressing the points above would further enhance its clarity, applicability, and impact.

**********

what does this mean?). If published, this will include your full peer review and any attached files.

If you choose “no”, your identity will remain anonymous but your review may still be made public.

**Do you want your identity to be public for this peer review?**  For information about this choice, including consent withdrawal, please see our Privacy Policy

Reviewer #1: **Yes:** S M Rashidul Hasan

Reviewer #2: No

**********

[NOTE: If reviewer comments were submitted as an attachment file, they will be attached to this email and accessible via the submission site. Please log into your account, locate the manuscript record, and check for the action link "View Attachments". If this link does not appear, there are no attachment files.]

To ensure your figures meet our technical requirements, please review our figure guidelines: https://journals.plos.org/plosone/s/figures

You may also use PLOS’s free figure tool, NAAS, to help you prepare publication quality figures: https://journals.plos.org/plosone/s/figures#loc-tools-for-figure-preparation.

NAAS will assess whether your figures meet our technical requirements by comparing each figure against our figure specifications.

# Author comment: Author response to Decision Letter 1

**DOI:** 10.1371/journal.pone.0355595.r002

8 May 2026

Responses to Reviewers

We sincerely thank the Editor and the Reviewers for their careful evaluation of our manuscript and for their thoughtful and constructive comments. We considered all suggestions with great attention and revised the manuscript accordingly. We believe that these revisions have significantly strengthened the paper. All changes in the revised version are highlighted in light blue. Below, we provide a detailed point-by-point response to each comment.

Reviewer #1

The manuscript addresses an important applied problem, uses an exceptionally large, nationally sampled data set, and combines appropriate analytic techniques (ROC analysis, latent profile analysis, and interpretable ML–based CAT). The methodological approach is coherent and the translational component (a Shiny application; code link on OSF) is commendable. However, several substantive issues must be addressed prior to publication to ensure conceptual accuracy, analytic robustness, and compliance with journal policies.

We sincerely thank the Reviewer for these positive comments and constructive suggestions. We greatly appreciate the recognition of the strengths of the study and have carefully considered all points raised in revising the manuscript.

1.The manuscript states the data are owned by the Italian National Institute of Health (Istituto Superiore di Sanità) and are not publicly available. This does not satisfy PLOS ONE’s data availability requirements. The authors must either:

a) deposit a suitably de-identified version of the dataset in a public or controlled-access repository with documented access procedures, or

b) provide a clear, formal controlled-access mechanism (repository name, contact, application procedure, and expected time to obtain access) that permits independent verification while protecting participant privacy, and include a justification that satisfies PLOS exception criteria. If legal/ethical constraints prevent sharing item-level data, the authors should provide synthetic data or aggregated tables sufficient to reproduce key results and supply all analysis scripts (complete R / Mplus code) together with the Shiny app configuration files.

We thank the Reviewer for this suggestion. A de-identified version of the dataset has been made publicly available on: https://osf.io/3857s/overview?view_only=8f89e81a29544c8fb5f9a3a92c531e06

2.The ROC cutoff is derived using a single self-report item about room confinement. That item is conceptually close to HRI content and therefore may bias classification (criterion contamination). The authors should:

Provide justification for using that single item as the external criterion and discuss its limitations explicitly; and, where possible, validate the cutoff against an independent clinical benchmark (e.g., structured clinical interview or clinician rating) or at least against external correlates (functional impairment, school absence).

Report ROC confidence intervals, decision thresholds sensitivity analyses (different dichotomizations), and calibration metrics.

We sincerely thank the Reviewer for this constructive comment. In the revised version of the manuscript, the rationale for using the room-confinement item as the external criterion has been better clarified and its limitations have been discussed more explicitly. In particular, the revised version of Introduction states more clearly that this item was selected because it captures a core behavioral manifestation of prolonged social withdrawal and extends previous HRI-15 work by relying on a more detailed, frequency-based criterion rather than on a simple binary indicator.

The ROC-related reporting has also been substantially expanded. Specifically, the revised manuscript reports confidence intervals for ROC performance indices, a sensitivity analysis based on a reasonable and more restrictive alternative dichotomization of the criterion (primary coding: responses 4–5 [“Every week, but not every day” / “Every day”] as positive; stricter coding: only 5 [“Every day”] as positive) and calibration diagnostics derived from logistic regression models estimated in the training set and applied to the testing set. These additions include confusion matrices, Brier scores, calibration intercepts and slopes, and calibration plots, reported in the main text and in the Supplementary Materials (main text: ROC Results section; Supplementary Tables S1–S5 and Figure S6).

In the revised Discussion, it is stated more explicitly that the external criterion remains a single self-report indicator rather than an independent clinical diagnosis, and that this may have introduced some degree of criterion misclassification, with possible implications for sensitivity, specificity, and the location of the optimal cutoff. The resulting threshold is therefore framed as a pragmatic screening indicator rather than as a definitive diagnostic benchmark.

Validation against an independent clinical benchmark (e.g., a structured interview or clinician rating) was not possible because such data were not available in the present survey. This limitation has been explicitly acknowledged, and future studies are indicated as needing to extend validation through clinical assessments, multi-informant information, and additional external indicators of functional impairment. It should nevertheless be noted that, although an independent clinical benchmark was unavailable, the observed associations between LPA-derived profiles, the external criterion, and ROC-based classification provided convergent support for the clinical meaningfulness of the identified threshold (Table 4).

3.The train/test split is appropriate but insufficient alone. Please provide one or more of the following robustness checks: repeated k-fold cross-validation, bootstrap validation, or external temporal/geographic validation (if available).

Justify hyperparameter choices for the conditional-inference tree (mincriterion/minsplit/minbucket). Provide sensitivity analyses showing how results (e.g., item savings, ICC, MAE) change when tuning these parameters.

Compare the proposed CART/ctree-based CAT with at least one alternative (for example: an IRT-based CAT, regularized regression, or random-forest surrogate) to demonstrate that the chosen approach balances interpretability and accuracy.

We really appreciate the comment from the Reviewer. In the revised version of the manuscript, the rationale for the selected ctree configuration has been clarified more explicitly, and the tuning results have been reported in greater detail. Specifically, candidate values for mincriterion, minsplit, and minbucket were examined over a broad grid of plausible settings, first through 5-fold cross-validation within the training subset and then through refitting on the full training set followed by evaluation on the independent test set. Moreover, it was further clarified that the selected configuration was retained because it provided the most appropriate balance between predictive accuracy and administration efficiency.

This allowed the sensitivity of agreement, prediction error, item savings, and tree complexity to alternative settings to be examined systematically. These additions are described in the main text (Method Section, Subsection “Machine-Learning Computerized Adaptive Test (CAT)”, Sub-subsection “CAT development”), and the detailed tuning and sensitivity results are reported in the Supplementary Materials (Tables S13–S16).

Although repeated k-fold cross-validation, bootstrap validation, or external temporal/geographic validation would certainly be valuable extensions, these analyses were not feasible within the scope of the present study. Nevertheless, robustness beyond a simple training/test split was addressed through internal cross-validation for model selection, independent test-set evaluation, and systematic sensitivity analyses across the examined hyperparameter grid.

A comparison with alternative approaches was also considered carefully. However, regularized regression and random-forest surrogates were judged to be less suitable as primary comparators because they do not naturally support intrinsically adaptive administration and are therefore not fully aligned with the main goal of the study, namely the development of an interpretable CAT for real-time adaptive administration. An IRT-based CAT would certainly represent an important benchmark and a highly relevant direction for future work; however, including such a comparison would have required introducing a substantially broader psychometric framework, with additional modeling assumptions and implementation choices, and would likely have moved the manuscript beyond its current focus. For this reason, the revision prioritizes a clearer justification of the selected ctree-based approach together with expanded robustness and sensitivity evidence.

4.The manuscript selects a four-class solution but reports significant VLMR/LMR p-values up to 5 classes. Provide a clearer, reproducible rationale for selecting four classes: include class stability checks (e.g., split-sample LPA), entropy and posterior probabilities per class, and substantive interpretability criteria.

Report class-specific sizes and standard errors clearly, and include the full fit-statistics table in supplement. Consider reporting model-based probabilities and provide a diagram/table with profile means and variance.

We sincerely thank the Reviewer for this important comment. In the revised version of the manuscript, the rationale for retaining the four-class latent profile solution has been clarified more explicitly and documented in greater detail. Specifically, the full fit-statistics table for the one- to five-class solutions is reported in the main text (Table 1), and the retained solution is justified on the basis of a joint evaluation of statistical fit, classification quality, class size, and substantive interpretability. Although the VLMR/LMR tests remained significant up to the five-class solution, the improvement from four to five classes was comparatively limited, whereas the four-class model showed the highest entropy and yielded a clearer and more interpretable configuration with well-defined class proportions. For this reason, the selected solution was framed as the best balance among fit improvement, classification quality, parsimony, and substantive interpretability. Additional classification diagnostics are reported in the Supplementary Materials, including estimated and modal class counts and proportions (Table S7), average posterior probabilities (Table S8), class-specific indicator means and standard errors (Table S9), within-class variance parameters (Table S10), and split-sample stability analyses across the training and testing subsets (Table S11 and Figure S12). These additions were intended to make the rationale for the retained four-class solution more transparent and reproducible.

5. Correct the in-text statement implying hikikomori is a formal DSM-5 diagnosis. The condition is a socio-cultural syndrome under scholarly debate and is not listed as a discrete DSM-5 disorder. Reframe language accordingly and cite current nosological discussions.

We sincerely thank the Reviewer for this comment. Based on this comment and on the relevant literature, the framing of hikikomori in the revised manuscript has been refined. In particular, the term is no longer presented as a formal diagnostic category in the DSM-5 or DSM-5-TR, but rather as a clinically relevant and culturally inflected syndrome/condition whose nosological status remains under scholarly debate. The revised text also specifies that hikikomori is mentioned in the DSM-5/DSM-5-TR in relation to cultural concepts of distress, but is not recognized as a discrete DSM-5/DSM-5-TR disorder. Current references addressing this nosological issue have also been added in the Introduction section.

6. Include confusion matrices, calibration plots, and calibration statistics for cutoff-based classification (both full-length and CAT-derived). Provide ICC tables with 95% confidence intervals.

For ML/CAT performance: report distributions of administered items, item-by-item visitation frequencies, and leaf-level prediction summaries (means, SD, n). Provide examples of cases where CAT and full-length scores diverged and explain why.

We thank the Reviewer for this comment. The requested classification and CAT-performance diagnostics have been substantially expanded in the revised version of the manuscript and in the Supplementary Materials. Specifically, confusion matrices, calibration statistics, and calibration plots are reported for both the full-length and CAT-derived classifications (full-length: Supplementary Tables S1–S5 and Figure S6; CAT-derived: Supplementary Tables S17–S19 and Figure S18). In addition, ICC values with 95% confidence intervals are reported for the agreement between CAT-derived and full-length scores (main text: Table 5; Supplementary Table S16 for the HRI-15 total score across tuning configurations).

With regard to CAT administration characteristics, the Supplementary Materials report the distribution of administered items (Supplementary Table S20 and Figure S21), item-by-item visitation frequencies (Supplementary Table S22), and leaf-level prediction summaries, which are provided in an Excel file on the OSF project, as indicated in the Supplementary Materials.

As for discrepancies between CAT-derived and full-length scores, the revised materials include a measurement-error-based summary of disagreement patterns across outcomes (Supplementary Table S23). Rather than adding a small set of illustrative single cases, an aggregate summary was preferred in order to avoid overinterpretation of potentially idiosyncratic observations. The reported results indicate that CAT–full-length discrepancies were generally limited across outcomes, as assessed through a measurement-error–based comparison using SEM-derived thresholds for meaningful score differences. A brief statement has therefore been added to the Supplementary Materials to clarify this point and to note that future research should examine more directly the conditions under which uncommon response patterns may yield larger CAT–full-length deviations.

Make all analysis code and the exact Shiny app tree/dictionary files available on the OSF project; include a README with instructions to reproduce the analyses and to run the Shiny app locally.

We thank the Reviewer for this suggestion. All analysis code, the Shiny app tree file, and related README files have been made available on OSF: https://osf.io/3857s/overview?view_only=8f89e81a29544c8fb5f9a3a92c531e06

Reviewer #2:

The manuscript presents a well-structured and methodologically rigorous study on the development of an interpretable machine learning–based computerized adaptive test (CAT) for hikikomori screening using the HRI-15. The topic is timely and relevant, and the integration of psychometric modeling with machine learning represents a meaningful contribution to both clinical assessment and large-scale screening.

A key strength of the study is the use of a large, nationally representative sample (N = 8,755), which provides strong statistical power and enhances the generalizability of the findings. The analytical framework is robust and appropriately implemented, combining ROC analysis, latent profile analysis (LPA), and a conditional inference tree–based CAT model. The use of a training/test split and multiple evaluation metrics (e.g., AUC, entropy, ICC, Cohen’s κ) demonstrates good methodological rigor and supports the validity of the conclusions.

The identification of a clinically interpretable cut-off score (≥ 42) and the derivation of four meaningful latent profiles represent valuable contributions, particularly for applied settings where both screening and personalization are needed. The CAT implementation is also well justified and shows substantial efficiency gains, reducing item administration while maintaining strong agreement with full-length scores. The inclusion of a Shiny-based application further enhances the practical utility of the work.

We sincerely thank the Reviewer for these thoughtful and constructive comments, and for the overall positive evaluation of the

Attachment Submitted filename: Responses to Reviewers.docx

# Peer review: Decision Letter 1

**DOI:** 10.1371/journal.pone.0355595.r003

**Authors:** Alberto Greco

23 Jul 2026

Interpretable Machine Learning for Hikikomori Screening: The Adaptive HRI-15

PONE-D-26-05328R1

Dear Dr. Anselmi,

We’re pleased to inform you that your manuscript has been judged scientifically suitable for publication and will be formally accepted for publication once it meets all outstanding technical requirements.

Within one week, you’ll receive an e-mail detailing the required amendments. When these have been addressed, you’ll receive a formal acceptance letter and your manuscript will be scheduled for publication.

An invoice will be generated when your article is formally accepted. Please note, if your institution has a publishing partnership with PLOS and your article meets the relevant criteria, all or part of your publication costs will be covered. Please make sure your user information is up-to-date by logging into Editorial Manager at Editorial Manager® and clicking the ‘Update My Information' link at the top of the page. For questions related to billing, please contact billing support.

If your institution or institutions have a press office, please notify them about your upcoming paper to help maximize its impact. If they’ll be preparing press materials, please inform our press team as soon as possible -- no later than 48 hours after receiving the formal acceptance. Your manuscript will remain under strict press embargo until 2 pm Eastern Time on the date of publication. For more information, please contact onepress@plos.org.

Kind regards,

Alberto Greco

Academic Editor

PLOS One

Additional Editor Comments (optional):

Reviewers' comments:

Reviewer's Responses to Questions

**Comments to the Author**

Reviewer #1: All comments have been addressed

**********

2. Is the manuscript technically sound, and do the data support the conclusions?

Reviewer #1: Yes

**********

3. Has the statistical analysis been performed appropriately and rigorously?

Reviewer #1: Yes

**********

4. Have the authors made all data underlying the findings in their manuscript fully available?

The PLOS Data policy

Reviewer #1: No

**********

5. Is the manuscript presented in an intelligible fashion and written in standard English?

Reviewer #1: Yes

**********

Reviewer #1: The revised manuscript has addressed all the concerns and is acceptable. The final version however, increase some readability, as in some cases, there are too many explanations. These can be either divided, or may be presented in the other ways, like table/figures, etc (as suits)

**********

what does this mean?). If published, this will include your full peer review and any attached files.

If you choose “no”, your identity will remain anonymous but your review may still be made public.

**Do you want your identity to be public for this peer review?**  For information about this choice, including consent withdrawal, please see our Privacy Policy

Reviewer #1: **Yes:** S M Rashidul Hasan

**********

# Editor report: Acceptance letter

**DOI:** 10.1371/journal.pone.0355595.r004

**Authors:** Alberto Greco

PONE-D-26-05328R1

PLOS One

Dear Dr. Anselmi,

I'm pleased to inform you that your manuscript has been deemed suitable for publication in PLOS One. Congratulations! Your manuscript is now being handed over to our production team.

At this stage, our production department will prepare your paper for publication. This includes ensuring the following:

* All references, tables, and figures are properly cited

* All relevant supporting information is included in the manuscript submission,

* There are no issues that prevent the paper from being properly typeset

You will receive further instructions from the production team, including instructions on how to review your proof when it is ready. Please keep in mind that we are working through a large volume of accepted articles, so please give us a few days to review your paper and let you know the next and final steps.

Lastly, if your institution or institutions have a press office, please let them know about your upcoming paper now to help maximize its impact. If they'll be preparing press materials, please inform our press team within the next 48 hours. Your manuscript will remain under strict press embargo until 2 pm Eastern Time on the date of publication. For more information, please contact onepress@plos.org.

You will receive an invoice from PLOS for your publication fee after your manuscript has reached the completed accept phase. If you receive an email requesting payment before acceptance or for any other service, this may be a phishing scheme. Learn how to identify phishing emails and protect your accounts at https://explore.plos.org/phishing.

If we can help with anything else, please email us at customercare@plos.org.

Thank you for submitting your work to PLOS ONE and supporting open access.

Kind regards,

PLOS ONE Editorial Office Staff

on behalf of

Dr. Alberto Greco

Academic Editor

PLOS One
